# Dissection of the T cell infiltrate in mouse pancreatic tumors reveals an extensive and diverse tumor-reactive T cell repertoire

**DOI:** 10.1126/sciadv.adr6132

**Published:** 2026-04-10

**Authors:** Hannes Kehm, Stefan Zens, Daniel Baumann, Zibo Meng, Arnoud H. de Ru, Rayman T. N. Tjokrodirijo, Caroline Vent, Olga Murawjew, Sarah Braun, Anne Weiss, Florian Bieberich, Aline Konrad, Francesca Lucato, Janne Kühner, Sonia Gutierrez Minguez, Chin Leng Tan, Jonas D. Förster, Mogjiborahman Salek, Angelika B. Riemer, Michael Volkmar, Peter van Veelen, Isabel Poschke, Rienk Offringa

**Affiliations:** ^1^ Division of Molecular Oncology of Gastrointestinal Tumors, German Cancer Research Center, Heidelberg 69120, Germany.; ^2^ Faculty of Biosciences, Heidelberg University, Heidelberg 69120, Germany.; ^3^ Department of General, Visceral and Transplantation Surgery, University Hospital Heidelberg, Heidelberg 69120, Germany.; ^4^ Sino-German Laboratory of Personalized Medicine for Pancreatic Cancer, Union Hospital, Tongji Medical College, Huazhong University of Science and Technology, Wuhan, Hubei 430022, China.; ^5^ Center for Medical Systems Biology, Leiden University Medical Center, Leiden, Netherlands.; ^6^ Faculty of Engineering, Heidelberg University, Heidelberg 69120, Germany.; ^7^ Division of Immunotherapy & Immunoprevention, German Cancer Research Center (DKFZ), Heidelberg 69120, Germany.; ^8^ Molecular Vaccine Design, German Center for Infection Research (DZIF), partner site Heidelberg, Heidelberg 69120, Germany.; ^9^ TCR Discovery Platform, Helmholtz-Institute for Translational Oncology by DKFZ (HI-TRON), Mainz 55131, Germany.

## Abstract

Although pancreatic cancer is generally refractory to immune checkpoint blockade, recent studies of tumor-infiltrating T cells in human tumor samples demonstrated the presence of in vivo expanded, tumor-reactive T cell receptor (TCR) clonotypes. Here, we explored the T cell repertoire in a murine pancreatic cancer model by combining single-cell transcriptomics with functional TCR characterization. This uncovered a substantial diversity of tumor-reactive TCR clonotypes. Whereas some of these were exclusively reactive against the autologous tumor, most TCRs reacted against syngeneic tumor cells of diverse tissue origin. Immunopeptidome analyses revealed three T cell epitopes reflecting distinct tumor antigen classes also found in human cancers: a mutanome-encoded neoantigen, an epitope encoded by an ectopically expressed endogenous retroviral provirus, and an epitope derived from a cell stress–induced autoantigen. These findings underline the importance of uncovering the antigen specificity of the natural tumor-reactive TCR repertoire to assess its therapeutic potential and safety with regard to personalized immunotherapy.

## Teaser

Pancreatic tumors harbor an extensive tumor-reactive T cell repertoire targeting different classes of tumor antigens.

## INTRODUCTION

Despite advances in surgical and cytostatic treatment, pancreatic ductal adenocarcinoma (PDAC) remains one of the deadliest cancer entities to date (*1*). The lack of effective treatment options is linked to key biological aspects of this disease, including the powerful combination of driver mutations, involving activation of the KRAS oncogene and loss of the tumor suppressor function of p53, CDKN2A, and/or SMAD4, as well as the barriers imposed by the tumor microenvironment (TME) (*2*, *3*). Consequently, the tumor is refractory to chemotherapy and targeted therapy. Furthermore, most pancreatic tumors are unresponsive to immune checkpoint blockade (ICB) (*4*, *5*). Nevertheless, we and others have found evidence for natural antitumor T cell immunity in human PDAC (*6*–*13*). More recently, we confirmed the presence of tumor-reactive (TR)-T cell receptor (TCR) clonotypes in the tumor-infiltrating T cell repertoire of these tumors by means of single-cell RNA sequencing (scRNA-seq) in combination with functional testing of TCRs for reactivity against autologous tumor cells (*14*). We found high frequencies of TR clonotypes in genetically unstable PDAC tumors, in line with the notion that patients with DNA damage repair–deficient PDAC may benefit from PD-1 (programmed cell death protein 1) antibody (Ab) treatment (*15*, *16*). We also identified TR clonotypes in regular PDAC tumor samples, thereby providing a perspective for personalized immunotherapy in this treatment-resistant indication.

In the present study, we performed an analogous analysis of the tumor-infiltrating T cell repertoire in a syngeneic, orthotopic PDAC mouse model with the aim to evaluate whether this could serve as a meaningful setting for preclinical evaluation of personalized immunotherapy modalities. This transplantable tumor (PDA30364), as previously described by us (*17*), originated from a primary pancreatic tumor in a mutant Kras/Trp53–driven genetically engineered mouse model (GEMM; Elas-tTA/TetO-Cre × LSL-Kras-G12D × LSL-Trp53-R172H). Like human PDAC, this tumor is refractory to ICB. Nevertheless, it can be efficiently controlled by combining MAPK (mitogen-activated protein kinase) kinase inhibitor (MEKi) treatment with agonist Abs targeting the immune stimulatory receptor CD40. Apart from the direct cytostatic impact of MEKi on the Kras-transformed tumor cells, the mechanism of action of this drug combination involved enhancement of the antitumor T cell response and reduction of the immunosuppressive myeloid cell infiltrate. T cell depletion diminished treatment efficacy to a partial suppression of tumor growth as observed upon treatment with MEKi alone, confirming the essential role of T cells in the therapeutic effect (*17*, *18*). We therefore dissected the infiltrating T cell repertoire in these tumors by means of scRNA-seq and functional TCR testing, with the aim to assess resemblances and differences with respect to our prior findings in human PDAC.

## RESULTS

### The T cell landscapes of both ICB-responsive and ICB-refractory pancreatic tumors reveal clusters with exhausted T cells

**Fig. 1.**
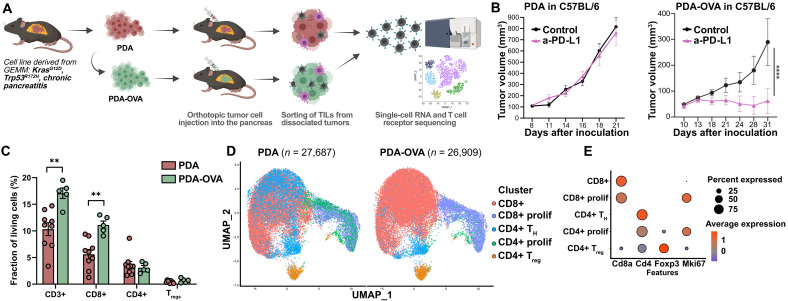
T cell landscape of ICI-refractory and ICI-responsive pancreatic tumors. (**A**) Schematic workflow toward the analysis of the tumor-infiltrating T cell repertoire in orthotopic pancreatic tumors (details in Materials and Methods). Tumor cell lines PDA30364 (hereafter PDA) and PDA-OVA (lentivirally transduced to express chicken OVA) were injected into the pancreas of syngeneic, immunocompetent C57BL/6 mice. Tumors were harvested and enzymatically dissociated into a single-cell suspension, followed by parallel scRNA-seq and TCR sequencing of sorted CD3+ T cells using the 10x Genomics platform. (**B**) Outgrowth of PDA tumors in C57BL/6 mice (*n* = 9 per group) treated with either anti–PD-L1 Ab (Atezolizumab, human IgG1) or isotype control Ab (200 μg, intraperitoneally) twice weekly (left). Same experiment as above for PDA-OVA tumors in C57BL/6 mice (*n* ≥ 13 per group, right). Two-way analysis of variance (ANOVA) with Sidak post hoc test; ****P < 0.0001. (**C**) Flow cytometric analysis of enzymatically dissociated tumors for the indicated T cell subsets, the sizes of which are expressed as the fraction of total living cells. T_regs_, T_reg_ cells. *n* ≥ 5, means ± SEM. Two-sided Student’s *t* test. **P < 0.005. (**D**) UMAP of 27,687 T cells from six PDA tumors and 26,909 T cells isolated from eight PDA-OVA tumors, clustered into major T cell subtypes. (**E**) Expression of T cell lineage markers displayed in a dot plot.

We investigated the nature of the T cell response in our pancreatic cancer tumor model (PDA30364, hereinafter referred to as PDA tumors) at the clonal level by means of scRNA-seq of flow cytometry–sorted CD3+ T cells (Fig. 1A). We used orthotopic tumors to approximate the TME of pancreatic cancer. As a positive control toward the identification of TR T cells, we also analyzed the T cell response in PDA-ovalbumin (OVA) tumors, which were generated by transducing the parental PDA tumor line with a gene construct encoding the traceable neoantigen chicken OVA (*17*). The PDA-OVA tumor line used herein was selected for low OVA expression levels that do not impede outgrowth in syngeneic, immunocompetent B6 mice (fig. S1, A to C). PDA-OVA tumors show increased T cell infiltration (fig. S1D) and, in contrast to the parental PDA tumor, are responsive to ICB using anti–programmed cell death 1 ligand 1 (PD-L1) Abs (Fig. 1B). Flow cytometric analysis of tumor-infiltrating CD3+ T cells shows that the PDA-OVA tumors are enriched in CD8+ T cells (Fig. 1C), a substantial fraction of which is reactive against the immunodominant H-2K^b^–restricted OVA/SIINFEKL T cell epitope (fig. S1E).

**Fig. 2.**
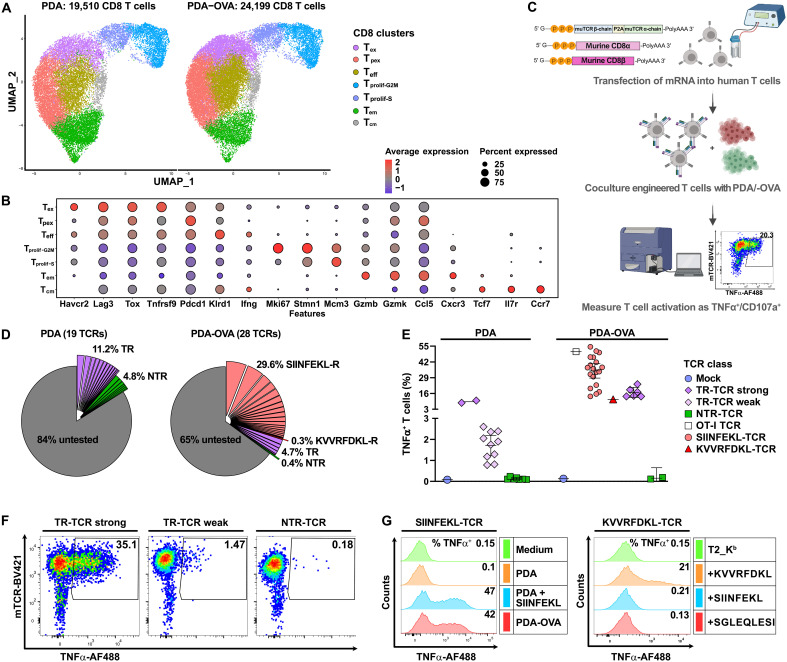
Identification of TR-TCRs in single-cell datasets from mouse pancreatic tumors. (**A**) UMAP of the CD8+ T cell subset from six parental PDA tumors (left) and eight PDA-OVA tumors (right). (**B**) Differentially expressed genes reflecting the seven main transcriptional states as identified in the aforementioned CD8+ T cell subset (see data S2 for complete gene lists). (**C**) Workflow for functional TCR screening involving the expression of tumor-derived TCRs along with murine CD8α/β in human T cells by means of mRNA transfection, followed by cocultivation with IFN-γ–pretreated tumor cells for 5 hours and measurement of T cell activation as intracellular flow cytometry staining for TNFα and/or CD107a. (**D**) Pie charts showing the functional activity of 19 expanded TCR clonotypes from PDA tumors, as well as 28 expanded clonotypes from PDA-OVA tumors. Percentages indicate the fraction of T cells with TR, OVA-reactive (SIINFEKL-R, KVVRFDKL-R), or NTR specificity from the T cell repertoires in PDA or PDA-OVA tumors. (**E**) Summary of the strength of the antitumor reactivity of the predominant CD8+ TCRs when expressed in human T cells that are cocultivated with PDA or PDA-OVA tumor cells. Shown is the reactivity of 12 tumor-associated antigen (TAA)–reactive TCRs (magenta) and 7 NTR-TCRs (green) as isolated from PDA tumors, as well as 19 OVA/SIINFEKL–specific TCRs (pink), 1 OVA/KVVRFDKL-specific TCR (red), 6 TAA-reactive TCRs (magenta), and 2 NTR-TCRs (green), as isolated from PDA-OVA tumors. TR-TCRs are grouped into strong, weakly, and nonreactive, as determined by intracellular staining for TNFα. All TCRs were tested at least two times with similar outcomes where mean values are displayed in the graph. (**F**) Example of flow cytometry data from a TCR screening experiment showing, from left to right, the TNFα production of T cells expressing a strong (CT2.5) or weak (CT4.2) TR-TCR and an NTR-TCR (CT3.1) when cocultured with PDA tumor cells. (**G**) Representative data showing the reactivity of an OVA/SIINFEKL–specific TCR (CT7.1, left) and a KVVRFDKL-specific TCR (CT10.4, right) against PDA, PDA-OVA, or human T2-Kb lymphoma cells, expressing murine H-2K^b^, that were pulsed with synthetic peptide epitopes.

Integration of the scRNA-seq datasets of CD3+ T cells from multiple PDA and PDA-OVA tumors created a UMAP (uniform manifold approximation and projection) landscape in which the T cells segregated into five primary transcriptional phenotypes: CD8+ cytotoxic and CD4+ T helper (T_H_) cells, their respective proliferating states, and CD4+ regulatory T cells (T_reg_ cells) (Fig. 1, D and E, and data S1). Zooming in on the CD8+ T cell subset, we found most T cells to locate in five UMAP clusters reflecting highly activated phenotypes, in particular the exhausted (T_ex_), preexhausted (T_pex_), effector (T_eff_), and proliferating (T_prolif-G2M_ and T_prolif-S_) states. The two further clusters reflect the effector memory (T_em_) and central memory (T_cm_)/naïve-like T cell states (Fig. 2, A and B; fig. S2, A and B; and data S2). Not only the ICB-responsive OVA-expressing tumors but also the ICB-refractory parental tumors showed prominent T_ex_ and T_pex_ clusters (fig. S2C), suggesting that functional analysis of the T cell infiltrate may reveal a high TR CD8+ T cell content in both tumor models.

### TR CD8+ TCR clonotypes were identified in scRNA-seq datasets of PDA tumors

We explored the presence of TR-TCR clonotypes in the CD8+ T cell landscape by means of in vitro functional testing of the corresponding TCRα/β pairs using a workflow that was successfully applied in our studies on the T cell response in human PDAC tumors (Fig. 2C) (*14*, *19*). As in our prior study, we performed an antigen-agnostic screen for reactivity against autologous tumor cells, rather than a screen against candidate mutanome-encoded neoantigens. The rationale for this was that our pancreatic cancer tumor model, like most human PDAC tumors, has a low mutational burden (data S3). Briefly, in vitro–transcribed mRNA encoding TCRα/β pairs was transiently transfected into human T cells, followed by coculture with tumor cells and measurement of T cell activation through intracellular cytokine staining for tumor necrosis factor–α (TNFα). The use of human T cells allows for selective detection of the transfected TCRs by means of a mouse TCR-Cβ–specific Ab while reducing mispairing with endogenous human TCR chains (*20*, *21*). To achieve the full functionality of mouse-derived TCRs, mRNA encoding the mouse CD8α and CD8β co-receptors was cotransfected (*19*).

The initial TCR screenings were unbiased with respect to the transcriptional profile of the TCR clonotypes, in that we focused on the TCRs expressed by the most expanded clonotypes. These analyses, including 47 TCRα/β pairs that were efficiently expressed (fig. S2D), resulted in the identification of TR-TCRs and non–tumor-reactive (NTR)-TCRs in both models (Fig. 2D). Of the 19 TCRs isolated from the ICB-refractory parental PDA tumors, 12 mediated antitumor T cell reactivity (Fig. 2, E and F). Expectedly, the fraction of TR-TCRs was even higher in the ICB-responsive PDA-OVA model (26 of 28) (Fig. 2E). Analysis of reactivity against an OVA-expressing B cell tumor line and against synthetic peptide epitopes revealed that most TR-TCRs isolated from the PDA-OVA tumors were directed against the immunodominant OVA/SIINFEKL T cell epitope. In addition, we found one TCR that was directed against a previously reported subdominant OVA/KVVRFDKL epitope (Fig. 2, E and G) (*22*). Nevertheless, six of the TR-TCRs isolated from OVA-positive tumors reacted against antigens distinct from OVA (Fig. 2E and fig. S2E). Together, these data suggest that the powerful OVA-specific T cell repertoire is instrumental in the ICB-induced rejection of PDA-OVA tumors (Fig. 1B), whereas the antitumor impact of the T cell response in the parental PDA model requires more potent regimens, such as the combination of MEK inhibition with agonist anti-CD40 Ab (*17*).

**Fig. 3.**
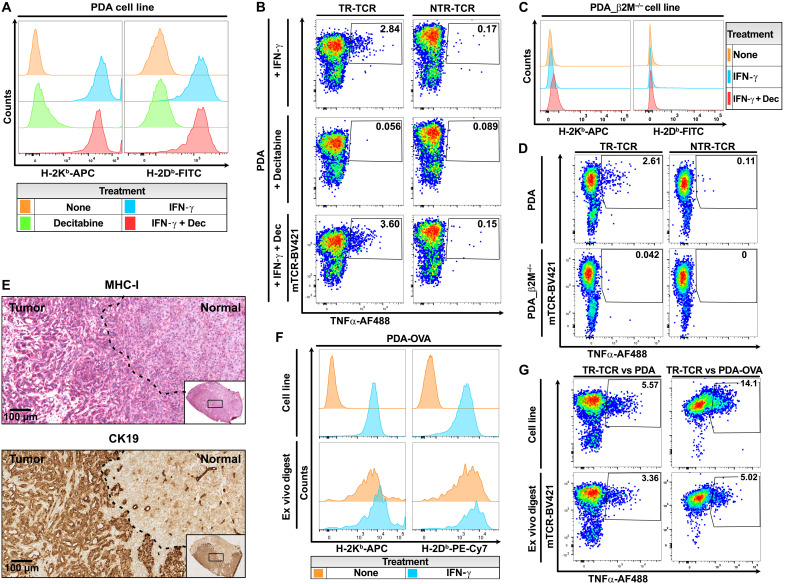
The TR T cell response is MHC restricted. (**A**) Cell surface expression of H-2K^b^ and H-2D^b^ on PDA cells under routine culture conditions or after 4-day treatment with IFN-γ and/or decitabine (Dec). (**B**) Impact of IFN-γ and/or decitabine pretreatment of PDA tumor cells on the responsiveness of human T cells expressing a weakly TR-TCR (CT3.3) versus an NTR-TCR (CT2.3). (**C**) Lack of H-2D^b^ and H2-K^b^ surface expression on β2M-knockout PDA tumor cells. (**D**) TNFα production of T cells expressing a TR-TCR (CT2.5, left) or an NTR-TCR (CT1.4, right) after coculture with either wild-type or β2M-deficient PDA tumor cells. (**E**) MHC class I staining on a PDA tumor section, as displayed by the Bond Polymer Refine Detection kit and hematoxylin (top). Cytokeratin-19 (CK19) staining on a serial tumor section, as displayed by DAB chromogen and alcian blue counterstain (bottom). The dashed line marks the border between tumor and healthy pancreatic tissue. The left half of the tissue section primarily consists of duct-like structures as shaped by tumor cells, the right half of untransformed acinar structures. Scale bar, 100 μm. (**F**) Flow cytometric analysis of H-2K^b^ and H-2D^b^ surface expression on the cultured PDA-OVA tumor cell line as compared to PDA-OVA tumor cells in freshly dissociated tumor tissue. The coexpression of OVA and GFP (green fluorescent protein) in PDA-OVA cells (see Materials and Methods) was used to set an unambiguous gate on the CD45^−^GFP^+^ tumor cells. MHC expression was analyzed without and with IFN-γ pretreatment. (**G**) T cell response of TR-TCRs (CT2.6 and CT9.6) against PDA/PDA-OVA tumor cell lines pretreated with IFN-γ/decitabine or dissociated tumor tissue.

We found that efficient detection of in vitro antitumor T cell reactivity mediated by especially the weaker, non–OVA-reactive TR-TCRs required pretreatment of the tumor cells with interferon-γ (IFN-γ) and the hypomethylating agent decitabine to increase major histocompatibility complex (MHC) class I cell surface expression and promote antigen presentation (Fig. 3, A and B). Both agents are known to improve T cell recognition of tumors by countering the silencing of genes encoding MHC class I proteins and components of the antigen processing machinery, as may happen in the course of in vivo tumor progression and in vitro propagation of cell lines (*23*–*28*). The specificity of the TCR-mediated reactivity against IFN-γ/decitabine–pretreated tumor cells was confirmed by the fact that no response was detected against tumor cells in which MHC class I surface expression was ablated through genetic knockout of the β2-microglobulin gene (Fig. 3, C and D). Nevertheless, the low constitutive MHC class I levels on cultured PDA tumor cells raised the question of whether these would present tumor antigens in vivo. Immunohistochemistry staining of the tumors showed prominent MHC class I expression by the tumor cells (Fig. 3E). The latter was confirmed by flow cytometric analysis of freshly dissociated tumors (Fig. 3F). This suggests that MHC expression is induced by interferons in the inflamed TME. Last, the tumor digests elicited TR-TCR–mediated T cell reactivity in vitro (Fig. 3G), further supporting the notion that the PDA tumor cells do present MHC class I–restricted antigens in vivo.

### TR and bystander T cells display distinct transcriptional states

**Fig. 4.**
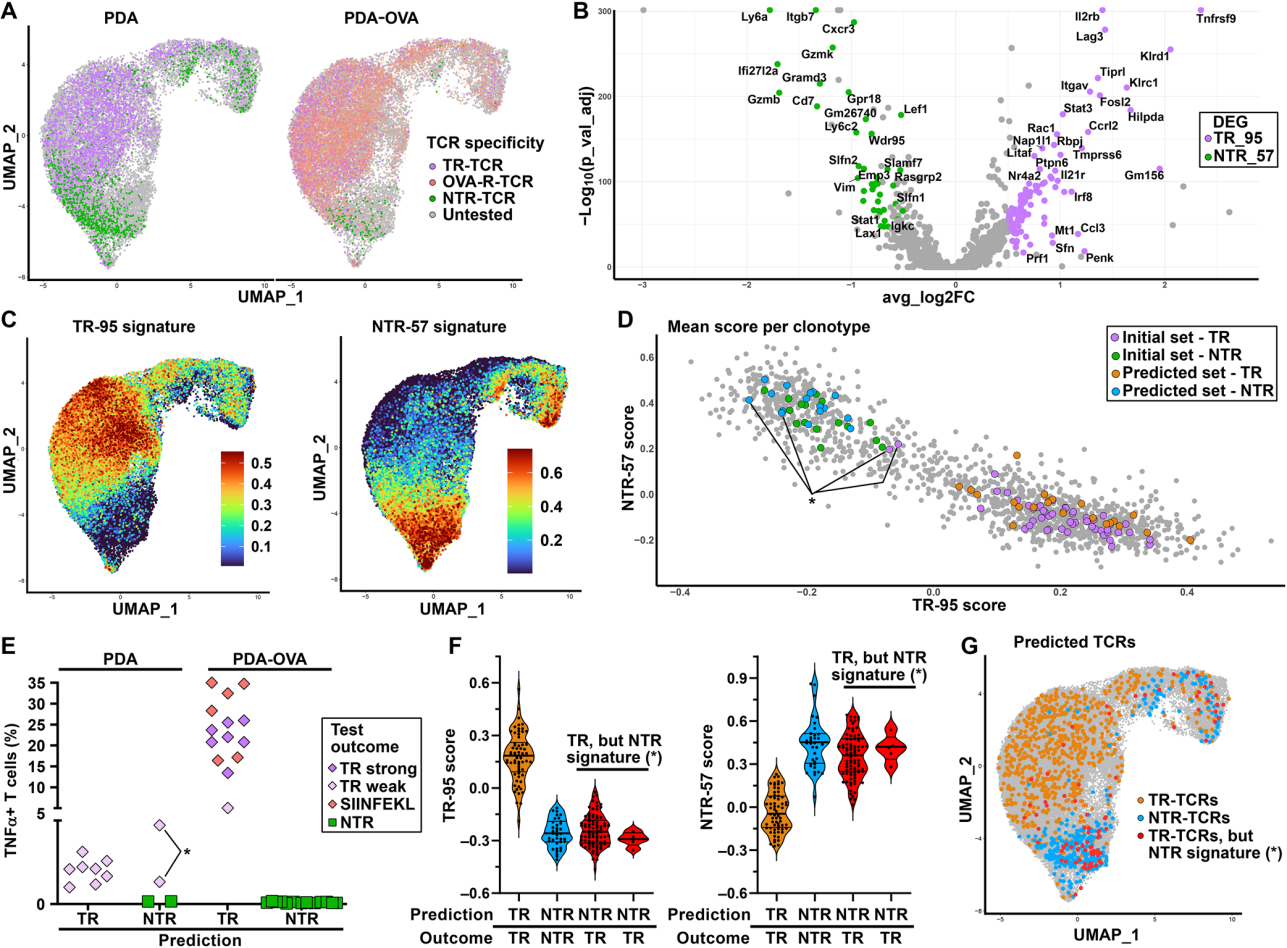
Delineation of gene signatures discriminating between TR and bystander T cells. (**A**) Projection of the individual T cells expressing 1 of the 47 tested TCRs by outcome in UMAP, showing the spatial segregation of T cells expressing TR-TCRs (magenta) and OVA-reactive TCRs (pink) compared to NTR-TCRs (green). (**B**) Volcano plot of genes differentially expressed between T cells included by validated TR-TCR and NTR-TCR clonotypes (data S4). DEG, differentially expressed gene. (**C**) UMAP depicting the TR-95 (left) and NTR-57 (right) scores as assigned to each individual CD8+ T cell on the basis of the expression of genes included in respective signatures using the AddModuleScore function in Seurat. (**D**) 2D matrix depicting the mean TR-95 and NTR-57 scores for each of the CD8+ TCR clonotypes included in the PDA/PDA-OVA datasets. Clonotypes with at least three cells are shown. Highlighted are the 47 clonotypes included in the first functional screen (magenta for TR and green for NTR), as well as the 39 further clonotypes included in the second screen predicted as TR (orange) or NTR (blue). The asterisk (*) marking indicates clonotypes that, on the basis of the gene signature, were predicted to be NTR but revealed antitumor activity in functional testing in vitro. (**E**) Summary of the antitumor reactivity of the 39 CD8+ TCRs selected on the basis of the gene signature. TCRs are grouped on the basis of predicted reactivity per model. (**F**) Violin plots showing the TR-95 (left) and NTR-57 (right) signature scores for individual T cells belonging to a typical TR clonotype (CT2.7, orange), a typical NTR clonotype (CT2.16, blue), and two unusual clonotypes (CT2.6 and CT2.82, red), the TCRs of which mediated antitumor reactivity in vitro in spite of being predicted to be NTR. The means ± 95% confidence interval are displayed. (**G**) Projection of the individual T cells expressing 1 of the 39 TCRs tested in the second screen in the UMAP of combined PDA and PDA-OVA CD8+ T cell data.

Projection of the functional TCR testing outcome onto the UMAP of the CD8+ T cell landscape revealed that T cells belonging to TR-TCR clonotypes are predominantly located in the T_ex_, T_pex_, and T_eff_ clusters (Fig. 4A). This indicates that the TR-TCR clonotypes as identified by our in vitro screenings also encounter their cognate antigens in the tumor in vivo. Projection of single TR-TCR clonotypes furthermore shows that most of the expanded TR-TCR clonotypes comprise cells in at least four transcriptional states: T_ex_, T_pex_, T_eff_, and T_prolif_ (fig. S3, A and B). This is reminiscent of our findings in human PDAC (*14*). In addition, small numbers of cells belonging to TR clonotypes can be found in the T_cm_/naïve-like cluster, which mainly encompasses T cells from nonexpanded TCR clonotypes (fig. S3C). These T cells most likely represent *Tcf7^+^Tox^−^* stem-like, tumor-specific memory cells that are rare in the TME and primarily reside in the tumor-draining lymph nodes (*29*–*32*). In contrast, the T cells belonging to NTR-TCR clonotypes primarily locate to the T_em_ cluster (Fig. 4A). The finding that also these NTR clonotypes are expanded and comprise T cells in the proliferating state (fig. S3D) suggests that these represent bystander T cell clones induced by recent pathogen encounters that have been attracted by the inflamed TME, as also observed in human PDAC tumors and other cancer types (*14*, *33*, *34*).

The gene expression programs associated with the TR-TCR versus bystander TCR clonotypes across all clusters were evaluated by means of differential gene expression analysis (Fig. 4B), resulting in the identification of 95 genes preferentially expressed by TR-TCR clonotypes (TR-95 gene set) and 57 genes preferentially expressed by NTR-TCR clonotypes (NTR-57 gene set) (see data S4 for the gene list and statistical thresholds applied). Whereas the TR-95 gene set comprises well-known markers associated with tumor reactivity such as *Tnfrsf9 (CD137, 4-1BB)* and *Lag3*, other typical markers such as *Havcr2* (TIM3), *Pdcd1*, *Tox*, and *Tigit*, although also preferentially expressed by T cells belonging to TR-TCR clonotypes, did not show statistically different levels in our gene expression analysis (fig. S4A). This higher overall degree of T cell activation, as compared to what is generally observed in tumor-infiltrating lymphocyte (TIL) scRNA-seq datasets of human tumors, is likely to reflect the fact that the tumors in transplantable mouse models arise within 2 to 3 weeks after injection of cells, which together constitutes a more immunogenic event than the stealthy, gradual development of most human cancers.

The UMAP feature plots in Fig. 4C depict the score of each individual CD8+ T cell using the gene signatures. The TR-95 gene set primarily highlights the T_ex_, T_pex_, and T_eff_ clusters, whereas the NTR-57 gene set essentially overlaps with the T_em_ cluster. Like in our human PDAC study (*14*), we applied the TR and NTR gene sets independently to calculate for each TCR clonotype a TR score and an NTR score, representing the mean scores for all T cells included. Integration of these data into a two-dimensional (2D) plot resulted in clear-cut segregation of the majority of already identified 38 TR clonotypes (purple dots) and 9 NTR clonotypes (green dots) into distinct areas (Fig. 4D and data S5).

### TR CD8+ TCR clonotypes can be identified on the basis of their gene signature

We applied the TR/NTR gene signature toward selection of 39 further CD8+ TCR clonotypes, 22 predicted to be TR (orange dots) and 17 predicted to be NTR (blue dots) (Fig. 4D). To create a balanced dataset of TR-TCR and NTR-TCR clonotypes for each of the tumor models, we skewed the selection toward TR-predicted clonotypes for the parental PDA tumors and NTR-predicted clonotypes for the PDA-OVA tumors. As shown in Fig. 4E, signature-based selection is highly specific, in that all (22 of 22) TCR clonotypes predicted to be TR were found to mediate antitumor T cell reactivity in vitro. As with the initial functional TCR screening, the overall strength of the TCRs from the OVA-positive tumors was higher, several of which were reactive against the OVA/SIINFEKL epitope. The TR-95 and NTR-57 signatures were also highly effective in identifying NTR/bystander TCR clonotypes (15 of 17), although in this context, we found two TCR clonotypes isolated from parental PDA tumors to mediate antitumor reactivity in vitro in spite of their in vivo NTR gene signature (Fig. 4, D and E, blue dots and purple diamonds, respectively, marked with asterisk). The same applies to two TCR clonotypes isolated from these tumors during the initial screening (Fig. 4D and fig. S4, B and C, purple dots marked with asterisks). Detailed analysis of the transcriptional state of these four TCR clonotypes at the single-cell level confirmed their close resemblance to bystander clonotypes (Fig. 4, F and G, and fig. S4D). This suggests that the optimized conditions of the in vitro assay, including the use of IFN-γ/decitabine–pretreated tumor cells, unveiled the antitumor reactivity of a small number of TCRs that do not show features of in vivo tumor antigen recognition, possibly due to limiting availability of their cognate antigen.

On the basis of the 86 functionally tested TCR clonotypes (fig. S4E), which cover 17.7 and 37.7% of the total T cell repertoire in the PDA and PDA-OVA scRNA-seq datasets, respectively**,** the TR/NTR gene signatures predicted TR-TCR clonotypes with a true positive rate of 100% (58 of 58) and a false-negative rate of 14% (4 of 28). The latter ambiguities were only observed in the less immunogenic PDA model (fig. S4, B and C). Receiver operating characteristic (ROC) analysis confirmed the slightly less optimal performance of the TR-95/NTR-57 signature in this model (fig. S4, F and G). This outcome reflects our findings in human PDAC tumors, in that signature-based discrimination between TR-TCR and NTR-TCR clonotypes was more clear-cut for the relatively immunogenic, genetically unstable PDAC samples than for the less immunogenic “regular” PDAC samples (*14*).

Using ROC analysis, we also evaluated the power of individual genes included in the TR and NTR gene sets with respect to discrimination between TR and bystander CD8+ T cells in the full dataset of tested TR-TCR and NTR-TCR clonotypes, thereby revealing the genes with the highest differentiating power (table S1 and data S6). These include, as noted above, well-known T_ex_/T_pex_ markers *Tnfrsf9* and *Lag3* but also less extensively studied T cell exhaustion markers such as the transcription factors *Rbpj* and *Nr4a2* (*35*, *36*). Whereas the latter genes are likely to contribute to the dysfunctional T_ex_ state, others may reflect further biological features of tumor-infiltrating T cells. For instance, the expression of *Hilpda* (hypoxia inducible gene 2; *Hig2*) reflects a metabolic survival strategy of cells exposed to hypoxic conditions (*37*). Expression of *Itgav* (alpha v integrin, CD51), as also found associated with T_ex_ in the context of other mouse tumor models and chronic lymphocytic choriomeningitis virus infection, was reported to enable rather than suppress the T cell function (*38*). As noted in the context of our prior study in human PDAC, the corresponding TIL gene signatures that were defined for different types of human cancers also comprise multiple genes for which the role in the T_ex_ state is not evident or clarified (*14*, *39*). Furthermore, we have noticed that differences between such gene signatures may reflect biological differences as well as aspects of technical/bioinformatic nature intrinsic to scRNA-seq (*14*). To further evaluate this with respect to mouse tumor models, we performed a comparative analysis between our TIL scRNA-seq dataset and the dataset that was created in the context of ProjecTILs on the basis of multiple TIL scRNA-seq datasets from MC38 and B16 tumors (*40*). Given that the studies resulting in these datasets did not include functional analysis of TCR clonotypes, we assessed to which extent the genes included in our TR-95 and NTR-57 signatures were preferentially expressed in the ProjecTILs T_ex_/T_pex_/T_eff_ clusters and ProjecTILs T_em_ cluster, respectively. This revealed that the majority of the most powerful [AUC (area under the curve) > 0.7] TR and NTR genes from our PDA model correctly segregated with these T cell states in the ProjecTILs dataset (table S1 and data S6 and S7).

Irrespective of the latter, the gene signatures that emerged from our studies in human PDAC samples and in the mouse PDAC tumor model differ considerably with respect to gene sets. Direct comparison of the mouse TR-95 signature with the corresponding TR signature that emerged from our studies of the human PDAC TIL repertoire shows that only 12 of the genes are conserved, including *Tnfrsf9*, *Lag3*, *Klrd1*, *Rbpj*, *Cxcr6,* and *Tnfrsf18* (data S6). As mentioned above, the interplay between tumor and T cell immune response is likely to differ between settings where solid tumors arise stealthily over the period of years in the context of an organ and settings where millions of tumor cells are injected into this organ, resulting in a progressively growing tumor within 2 to 3 weeks. Even though TR/NTR signatures do not fully translate between mouse models and human cancers, both are highly effective in selecting TR-TCR versus NTR-TCR clonotypes in the respective biological settings concerned.

### Gene signature–based analysis also identifies TR CD4+ TCR clonotypes

**Fig. 5.**
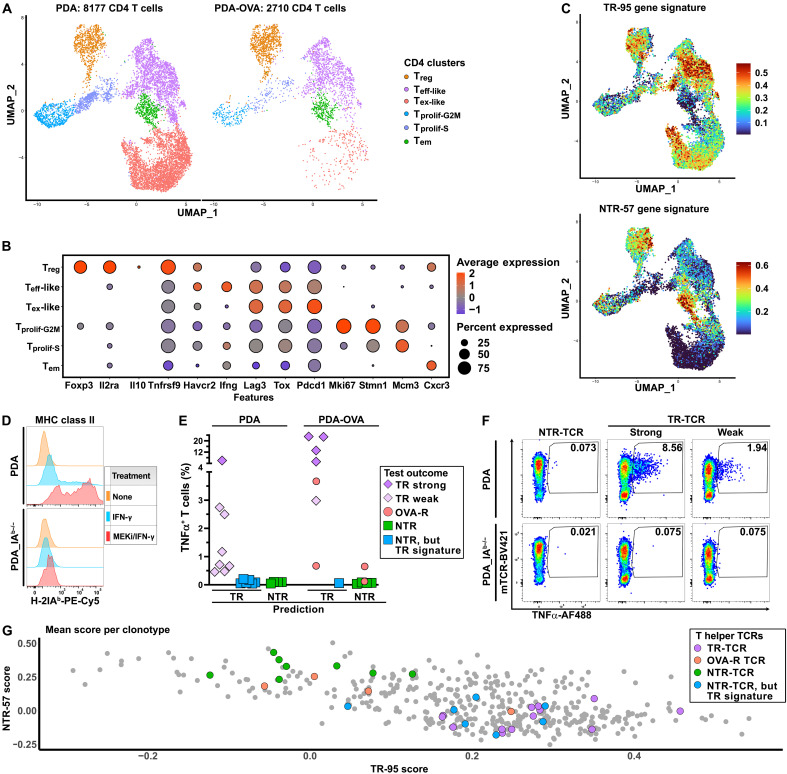
Dissection of MHC class II–restricted CD4+ T cell immunity in murine PDA tumors. (**A**) UMAP of the CD4+ T cell subsets from PDA and PDA-OVA tumors. (**B**) Differentially expressed genes reflecting the main transcriptional states as identified in the aforementioned CD4+ T cell subset (see data S8 for the complete gene lists). (**C**) UMAP depicting the TR-95 (top) and NTR-57 (bottom) scores as assigned to each individual CD4+ T cell on the basis of the expression of genes included in respective signatures using the AddModuleScore function in Seurat. (**D**) Cell surface expression of MHC class II I-A^b^ on wild-type or I-A^b^–deficient PDA cells cultured under routine conditions or after treatment with IFN-γ and/or MEKi GDC-0623. (**E**) Summary of the antitumor reactivity and antigen specificity of 32 tested CD4+ TCRs from PDA/PDA-OVA tumors (18 TCRs from PDA and 14 further TCRs from PDA-OVA) when expressed—together with the mouse CD4 co-receptor—in human T cells that are cocultivated with PDA/PDA-OVA tumor cells pretreated with IFN-γ/MEKi. Shown is the reactivity of 13 TR-TCRs (magenta) predicted to be TR based on the gene signature (eight PDA and five PDA-OVA). The square icons represent 15 NTR-TCRs that, on the basis of their gene signature, were predicted to be NTR (green; four PDA and four PDA-OVA) or TR (blue; six PDA and one PDA-OVA). An additional four TR-TCRs from PDA-OVA (pink circles) detect OVA-derived antigen [see fig. S5 (D to G)]. (**F**) Representative data of functional TCR screening experiments showing, from left to right, the TNFα production of T cells expressing TCRs derived from PDA tumors that mediate either no (CT4.3), strong (CT6.3), or weak (CT5.1) T cell reactivity against either wild-type or I-A^b^–deficient PDA tumor cells. (**G**) 2D matrix depicting the mean TR-95 and NTR-57 scores of the CD4+ clonotypes included in the PDA/PDA-OVA datasets. Clonotypes with at least three cells are shown. Highlighted in different colors are the 32 functionally tested clonotypes (magenta for TR, pink for OVA-reactive, green for NTR clonotypes, and blue for NTR clonotypes with a TR gene signature).

Given the importance of the CD4+ T cell response in antitumor immunity, also in solid tumors that do not constitutively express MHC class II (*41*, *42*), we zoomed in on the CD4+ T cell subsets of the scRNA-seq T cell landscape (Fig. 1, D and E). This revealed a landscape featuring four clusters with highly activated T cells. Although the corresponding T cell states do not exactly reflect those identified in the CD8+ T cell landscape, we could distinguish a T_eff_-like cluster characterized by the expression of *Ifng*; a T_ex_-like cluster characterized by higher expression of *Lag3*, *Tox*, and *Pdcd1*; and proliferating G_2_-M and S phase clusters (Fig. 5, A and B, and data S8). These were more prominent in the PDA tumors, probably due to the absence of the highly immunogenic OVA/SIINFEKL CD8+ T cell epitope. As shown in fig. S5A, especially the T_ex_-like cluster harbors the highest density of expanded clonotypes. We therefore explored whether our CD8+ T cell–based TR/NTR gene signature could also be used to identify TR CD4+ TCR clonotypes. The TR signature highlights the T_ex_-like and T_eff_-like clusters, whereas the NTR signature marks a distinct T_em_-like cluster (Fig. 5C), suggesting that these harbor TR and bystander clonotypes, respectively. The CD4+ T cell landscape furthermore comprises a *Foxp3+* T_reg_ cluster, which is highlighted by both the TR and NTR signatures because of coexpression of T_ex_/T_pex_–related and T_em_-related marker genes (e.g., *Tnfrsf9* and *Cxcr3*; see Fig. 5B and data S8). As for the cytokine gene profile of the CD4+ T cell response, *Ifng* expression was prominently detected in the T_eff_-like state and, to a lesser extent, in the proliferating and T_em_ states (fig. S5B). Expression of *Il10* was predominantly detected in the T_reg_ cluster, whereas our datasets showed only marginal expression of *Il4*, *Il5*, and *Il17* (fig. S5C and data S8). Together, also taking into consideration the CD8+ T cell landscape (Fig. 2, A and B) and the notion that these tumors are rich in M_2_-type macrophages and myeloid-derived suppressor cells (*17*, *18*), this renders the picture of a strong CD4+T_H_1/CD8+ antitumor response that is blunted by CD4+ T_reg_ cells and the myeloid cell infiltrate.

On the basis of this initial survey, we sampled the tumor-infiltrating CD4+ T cell repertoire by cloning the TCRs from 22 expanded T_H_ clonotypes with a prominent TR gene signature and strong representation in the T_eff_-like and T_ex_-like clusters. In addition, we isolated TCRs from 10 expanded clonotypes with NTR signatures located in the T_em_ cluster, expecting these to not mediate antitumor reactivity. For functional testing of CD4+ T cell–derived TCRα/β pairs, we applied the same workflow as shown in Fig. 2C, with the difference that mRNA encoding the mouse CD4 co-receptor was cotransfected to achieve the full functionality of mouse-derived TCRs (*19*). Furthermore, we exploited our previously reported finding that combined pretreatment of the tumor cells with IFN-γ and MEKi results in strong up-regulation of the I-A^b^ MHC class II molecule at the cell surface (Fig. 5D and fig. S5D) (*17*). Like for the CD8+ T cell subset, this screen resulted in the identification of multiple CD4+ TR-TCRs in both tumor models (Fig. 5E). Also, in the CD4+ dataset, the TCRs showed different strengths. Even the weakly reactive TCRs displayed bona fide MHC class II restriction, in that these failed to mediate T cell responses against I-A^b^-knockout tumor cells (Fig. 5F). As discussed above with respect to MHC class I–restricted antigen presentation, the detection of TCR reactivity against IFN-γ/MEKi–pretreated tumor cells in vitro raises the question of whether MHC class II–restricted antigen presentation by tumor cells would also take place in vivo. As shown in fig. S5D, a fraction of tumor cells in freshly dissociated tumor samples does express MHC class II, but the levels are markedly lower than those on the IFN-γ/MEKi–pretreated tumor cells. In view of this, the activated state of the TR CD4+ TCR clonotypes may primarily be induced by professional antigen-presenting cells, such as dendritic cells or tumor-infiltrating macrophages that have taken up and processed exogenous tumor antigens, rather than through direct antigen presentation by the tumor cells.

In spite of the fact that most TR CD4+, MHC class II–restricted TCR clonotypes could be identified correctly on the basis of the TR/NTR gene signatures, the prediction was less accurate than for CD8+ T cell clonotypes. In particular, the TCRs from seven clonotypes with a prominent TR signature did not mediate tumor cell recognition in vitro (Fig. 5, E and G, blue icons, and data S9). Also, for this paradox, the indirect presentation of tumor-derived antigens by professional antigen-presenting cells offers a plausible explanation. Direct presentation of MHC class II–restricted epitopes by tumor cells and other nonprofessional antigen-presenting cells is an understudied subject (*43*), but it is evident that the antigen processing mechanisms involved in this direct presentation are more limited than those available in professional antigen-presenting cells (*44*). In view of this consideration, it is highly conceivable that the in vivo TR gene signature of these CD4+ TCR clonotypes reflects their active engagement in the antitumor immune response through encounter of their cognate antigen on professional antigen-presenting cells. This notion is supported by the distribution of T cells belonging to these clonotypes in the T_ex_-like/T_eff_-like clusters (fig. S5E). The difference between in vivo and in vitro MHC class II–restricted antigen presentation is further illustrated by the four TCRs as isolated from PDA-OVA tumors that display in vitro reactivity against OVA-expressing tumor cells, in that two of the corresponding clonotypes displayed a clear-cut NTR gene signature [Fig. 5, E and G, pink icons; see fig. S6 (A to C) for clonotype-specific details]. For these TCRs, enhancement of antigen presentation by IFN-γ/MEKi pretreatment of the PDA-OVA cells allowed the detection of their cognate antigens in the in vitro assay. Notably, these MHC class II–restricted, OVA-reactive TCRs mediate strong T cell reactivity against an OVA-expressing B cell tumor line (fig. S6D). Irrespective of the aforementioned considerations, it is evident that the CD8+ T cell–based TR/NTR gene signature is less effective in predicting the antitumor reactivity of CD4+ TCR clonotypes.

### Analysis of TR-TCR clonotypes revealed distinct reactivity patterns

**Fig. 6.**
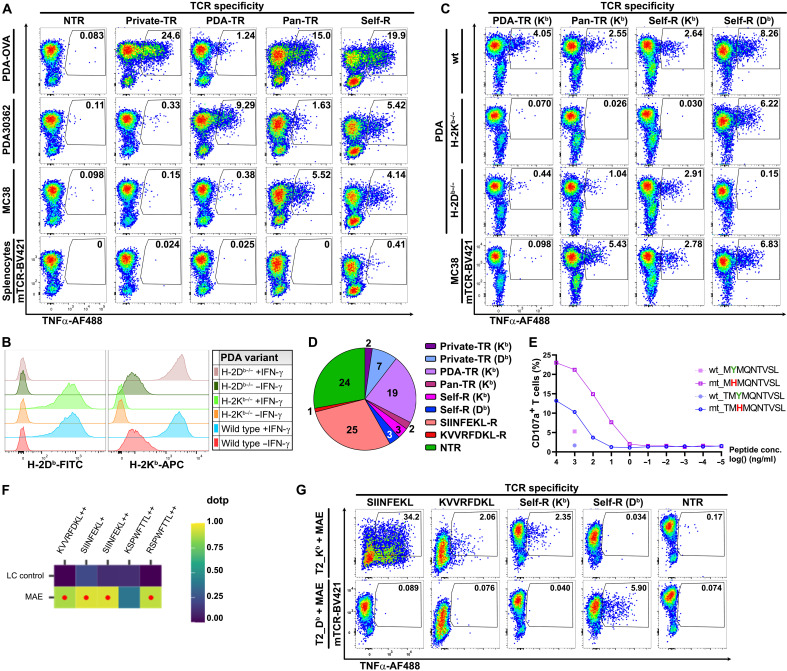
Differentiation between CD8+ TR-TCRs based on tumor reactivity, MHC restriction, and antigen specificity. (**A**) Representative TCR screening data showing TNFα production of T cells expressing different classes of TR-TCRs. From left to right: An NTR-TCR (CT12.37); a private TR-TCR (CT11.2) reactive to PDA-OVA only; a PDA-reactive TCR (CT2.5) reactive against PDA, PDA-OVA, and PDA30362; a pan-TR-TCR (CT14.2) also responding to MC38 cells; and a self-R TCR (CT11.10) detecting a self-antigen expressed by cultured C57BL/6 mouse splenocytes. See fig. S7A for T cell reactivity in the same experiment on the basis of CD107a. (**B**) Selective deficiency of H-2D^b^ or H2-K^b^ expression on PDA tumor cells, as induced by CRISPR-Cas9. (**C**) Reactivity of representative TCRs of different classes (from left to right: CT2.5, CT14.2, CT2.6, and CT11.10) against MC38 tumor cells, as well as against PDA tumor cells selectively knocked out for H-2K^b^ or H-2D^b^. wt, wild type. (**D**) Overview of the six classes of TR-TCRs targeting TAAs, as isolated from 86 T cell clonotypes, subclassified on the basis of MHC restriction. The number of identified TCRs per class is indicated (data S10). (**E**) Detection of the Hook3 nona/decamer (T)MHMQNTVSL by TR-TCR CT14.7 when loaded at different concentrations onto T2-D^b^ cells. Dashed lines indicate reactivity against the corresponding wild-type variants (1 μg/ml). (**F**) Overview of peptides detected in mild acid eluate from PDA-OVA cells through targeted MS. The dot product (dotp) color code reflects the similarity of the detected peptide-fragmentation patterns to the high-resolution MS2 spectral library generated for these peptides at the indicated charge states (+ or ++ stands for H+ or 2H+, respectively) by Prosit. Red dots indicate plausible detection (dotp ≥0.85). (**G**) TNFα production of T cells expressing TCRs with the indicated antigen/tumor specificity (from left to right: CT7.1, CT10.4, CT2.6, CT11.10, and CT7.6). The T cells were incubated with T2-K^b^ or T2-D^b^ cells pulsed with MAE peptide fractions.

Together, the functional TCR screenings resulted in the characterization of 118 TCRs, derived from 86 CD8+ and 32 CD4+ tumor-infiltrating T cell clones, of which 49 TCRs mediate reactivity against the pancreatic tumors through non-OVA T cell epitopes derived from endogenously expressed tumor antigens (data S10). To facilitate identification of such epitopes by means of immunopeptidomics, we first classified these TCRs on the basis of reactivity to different tumor cell lines of H-2^b^ mouse origin, as well as for MHC restriction. In view of technical feasibility, especially the identification of epitopes based on fixed length, we focused on TCRs derived from CD8+ T cell clones. On the basis of tumor cell recognition, we identified four categories of TR-TCRs (Fig. 6A and fig. S7, A and B): First, we found “private” TCRs exclusively reactive against the tumor of origin. Second, we identified “PDA-reactive” TCRs that also responded against the PDA30362 cell line, which like PDA30364 originated from a tumor that arose in an Elas-tTA/TetO-Cre × LSL-Kras-G12D × LSL Trp53-R172H GEMM mouse (a littermate) within the same experiment. Third, we found “pan-TR” TCRs, which in addition mediated T cell reactivity against the immuno-oncology workhorse tumor lines MC38 and B16 of C57BL/6 mouse origin. Last, we identified autoreactive TCRs that in addition reacted against normal syngeneic splenocytes. Notably, the latter reactivity is barely detectable above the background when TNFα is used as a readout for T cell activation but clearly noticeable when flow cytometric detection of CD107a surface expression is used (fig. S7, A and B). In the course of our work, we found that this marker, reflecting degranulation of CD8+ T cells, provides an overall more sensitive readout for the functional screening of MHC class I–restricted TCRs as expressed by CD8+ T cell cultures (*19*). The availability of PDA cells selectively knocked out for either H-2K^b^ or H-2D^b^ allowed us to further classify the TCRs on the basis of MHC restriction (Fig. 6, B and C). Together, this divided the TCRs detecting tumor antigens other than OVA-derived epitopes into six classes (Fig. 6D).

### Deorphanization of TR CD8+ TCRs identified three different target antigens

The TCR reactivity patterns and MHC restrictions observed suggested that the K^b^-restricted pan-TR-TCRs might be directed against the previously identified p15E (KSPWFTTL) and Cripto-1 (SAFEFGPVA) epitopes. These epitopes, respectively derived from an endogenous retroviral antigen (the env protein p15E) and a tumor-associated autoantigen, were reported by others to be presented by MC38, B16, and mouse pancreatic tumor cell lines (*45*–*47*). However, none of the TCRs concerned showed reactivity against antigen-presenting cells pulsed with these peptides. Nevertheless, we did find that several TR-TCRs responded to synthetic peptide pools with fixed anchor residues for binding to either H-2K^b^ or H-2D^b^ in a manner that matched their MHC restriction, as identified by means of PDA H-2D^b^/K^b^ knockout cells (fig. S7C). Notably, we made the same observation for several NTR-TRs isolated from bystander TCR clonotypes, illustrating that also these TCRs are functionally expressed. This is expected, because in humans, expanded bystander clonotypes have been shown to react against common pathogens such as CMV (cytomegalovirus), EBV (Epstein-Barr virus), and influenza virus (*14*, *33*, *34*), while transfer of SPF (specific pathogen–free)–bred mice to conventional animal housing for experimentation is known to result in the induction of immune responses as a result of microbial exposure (*48*, *49*).

In the context of the current interest in therapeutic exploitation of the neoantigen-specific T cell response in pancreatic cancer (*9*, *50*), the detection of reactivity by “private” TCRs in this experiment prompted us to search for potential tumor mutanome-encoded neoepitopes. Combined DNA/RNA sequencing analysis revealed 98 mutated sequences involving a nonsynonymous single-nucleotide variant or frameshift, 60 of which were expressed at the mRNA level (data S3). Subsequent prediction of H-2D^b^/K^b^–binding neoepitopes by means of the NetMHCpan-4.1 algorithm (*51*) identified 22 candidates (data S11). Analysis of the corresponding synthetic peptides for recognition by H-2D^b^/K^b^–restricted “private” TCRs revealed that one of the seven H-2D^b^–restricted TCRs responded against a peptide with the sequence (T)MHMQNTVSL, comprising a Y-to-H substitution at amino acid position 2 (Fig. 6E and fig. S8A). This epitope is derived from Hook microtubule-tethering protein 3 (Hook3) (*52*), an adapter protein involved in intracellular trafficking for which inactivating mutations have been reported in different human cancers. Both the 9- and 10-mer variants of the mutated peptide are recognized, with the 9-mer eliciting stronger T cell activation, whereas the wild-type counterparts only elicited very weak T cell reactivity at ≥100-fold higher concentrations, in spite of also being strong D^b^-binders (data S11). Accordingly, the amino acid substitution is located in the N-terminal portion of the peptide and does not affect the main anchor residues, being the central asparagine and C-terminal leucine (*51*).

In parallel, we tested TCR reactivity against the natural peptidome of PDA-OVA cells as isolated by means of a mild acid elution protocol (*53*), which enabled technical validation through detection of the known OVA-derived epitopes by means of targeted mass spectrometry (MS) (Fig. 6F and fig. S8B) (*54*–*56*). The aforementioned p15E epitope was also readily detected, albeit the RSPWFTTL variant (K-to-R substitution of the first residue) that results from polymorphisms in the endogenous retroviral sequences concerned. Notably, our transplantable PDA model originated from a GEMM model that, although backcrossed into C57BL/6, harbors genetic remnants of the 129/Sv founder strain (*17*). The p15E/RSPWFTTL epitope was found to lack immunogenicity in B6 mice because of central tolerance (*57*). This explains why this epitope—although presented abundantly—does not play a role in the natural T cell response in our PDA model. In line with the MS data, the peptide preparation elicited antigen-specific T cell reactivity when presented to T cells expressing TCRs targeting either of the OVA epitopes (Fig. 6G). This screening also revealed the presence of peptides that were recognized by self-reactive TCRs, again in line with their predefined MHC restriction.

**Fig. 7.**
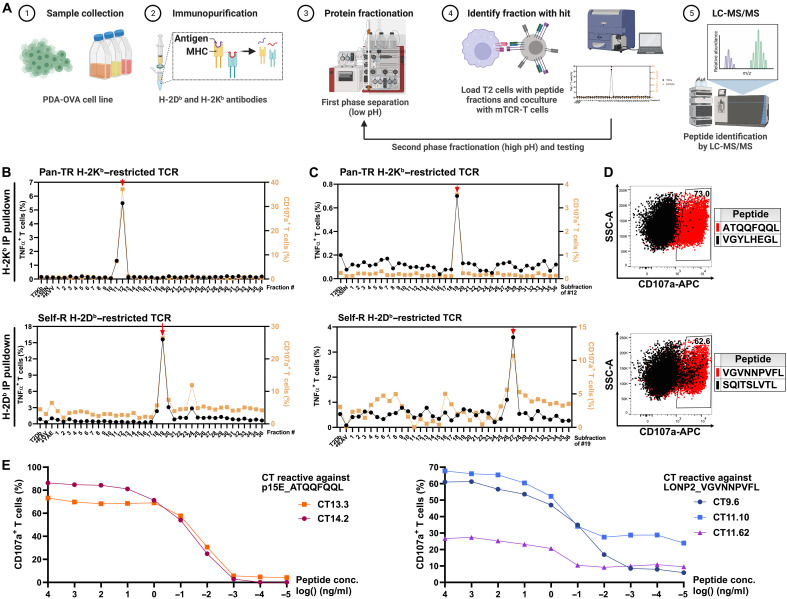
Identification of two tumor-associated epitopes by means of immunopeptidomics. (**A**) Schematic workflow toward epitope identification of TR-TCRs against peptides eluted from H-2K^b^/H-2D^b^. Peptide preparations from immunopurified H-2K^b^ and H-2D^b^ molecules of PDA-OVA cells were HPLC fractionated over two consecutive gradients at different pH values. Fractions obtained from the first gradient were loaded onto T2-K^b^ or T2-D^b^ cells, followed by cocultivation with T cells expressing a TCR of interest. T cell activation was measured by detection of TNFα and CD107a. In case one or more positive fractions were identified, these—along with bordering negative fractions—underwent secondary fractionation, after which functional screening was repeated. In case again positive fractions were identified, these—along with bordering negative fractions—were resolved by means of LC–tandem MS (LC-MS/MS), followed by evaluation of the identified masses in the context of the reference proteome. (**B**) Screening outcome of primary HPLC fractions with T cells expressing the TCRs of pan-TR H-2K^b^–restricted CT14.2 (top) and self-R H-2D^b^–restricted TCR CT9.6 (bottom). IP, immunoprecipitation. (**C**) Screening outcome of the secondary HPLC fractions for the two aforementioned TCRs, resulting in the identification of positive secondary fractions that were subsequently analyzed by means of LC-MS/MS. (**D** and **E**) Validation of the T cell epitopes as identified by LC-MS/MS in the positive secondary fractions by means of synthetic peptide analogs. The TCRs of pan-TR H-2K^b^–restricted CT14.2 (top) and self-R H-2D^b^–restricted TCR CT9.6 (bottom) react against the p15E-derived epitope ATQQFQQL and the LONP2-derived epitope VGVNNPVFL, respectively, but not against control peptides as identified by LC-MS/MS in the same secondary fractions (respectively VGYLHEGL, an H2-K^b^–binding peptide derived from U5 small nuclear ribonucleoprotein 200-kDa helicase, a spliceosome subunit, and SQITSLVTL, an H2-D^b^–binding peptide derived from origin recognition complex subunit 5, a protein involved in DNA replication). Synthetic peptides were pulsed onto T2-K^b^ or T2-D^b^ cells at 1 μg/ml.

Encouraged by these findings, we embarked on the identification of peptide epitopes at the molecular level by means of tandem MS in high-performance liquid chromatography (HPLC)–fractionated immunopeptidome preparations as isolated from immunopurified MHC molecules (Fig. 7A). TCR screening of primary fractions resulted in positive signals for four different classes of TR-TCRs, as well as for the TCRs directed against the OVA/SIINFEKL T cell epitope (Fig. 7B and fig. S9A). After secondary fractionation, positive signals were maintained for three TR-TCR classes, as well as for TCRs targeting the OVA/SIINFEKL epitope (Fig. 7, B and C, and fig. S9A). Systematic comparison of the MS data of the peak and bordering fractions in the context of the reference proteome (data S12) subsequently revealed, in addition to the OVA/SIINFEKL peptide, two tumor-associated epitopes, the TR-TCR–mediated recognition of which was experimentally confirmed by means of synthetic peptide analogs (Fig. 7, D and E, and fig. S9, B and C). One of the epitopes (ATQQFQQL), recognized by two of the two H2-K^b^–restricted pan-TR-TCRs, was found to be derived from the endogenous p15E retroviral antigen. This epitope is distinct from the aforementioned retroviral p15E/KSPWFTTL antigen (*58*). In line with the pan-tumor reactivity of these TCRs, this epitope was recently identified by others in the B16 model (*59*, *60*). The second epitope (VGVNNPVFL), recognized by three of the three H2-D^b^–restricted self-reactive TCRs, is derived from peroxisomal Lon protease LONP2. Lon proteases play an important role in maintaining the function of peroxisomes (LONP2) and mitochondria (LONP1) in the context of cellular oxidative stress by degrading proteins damaged by oxidation. Elevated LONP expression has been observed in different human cancers, whereas LONP deficiency was shown to suppress tumor cell growth (*61*). The fact that LONP2 also plays a role in peroxisomal protein homeostasis in nontransformed cells offers a plausible explanation for the reactivity of the corresponding self-reactive TR-TCRs against nontransformed splenocytes. For selected TCRs representing the different reactivity patterns, we performed experiments using retrovirally transduced primary mouse T cells (fig. S10). These experiments reproduced the results obtained with mRNA-transfected human T cells.

### The TR-TCR repertoires in PDA and MC38 tumors show partial overlap regarding antigen specificity

**Fig. 8.**
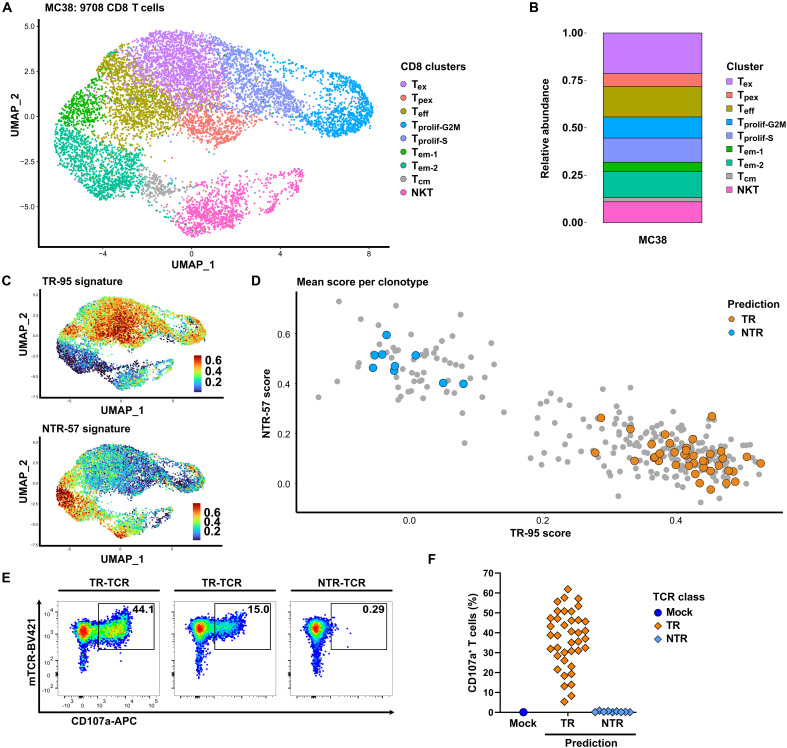
Identification of TR-TCRs in single-cell datasets from MC38 tumors. (**A**) UMAP of the CD8+ T cell subset from three MC38 tumors. NKT, NK T cell. (**B**) Relative abundance of T cells in the different transcriptional states. See fig. S11 for UMAP of total T cell landscape and for differentially expressed genes reflecting the transcriptional states as identified in the total and CD8+ T cell landscapes. (**C**) UMAP depicting the TR-95 (top) and NTR-57 (bottom) scores as assigned to each individual CD8+ T cell on the basis of the expression of genes included in respective signatures using the AddModuleScore function in Seurat. (**D**) 2D matrix depicting the mean TR-95 and NTR-57 scores for each of the CD8+ TCR clonotypes included in the MC38 dataset. Clonotypes with at least three cells are shown. Highlighted are the 48 clonotypes included in the functional screen (orange for TR and blue for NTR). (**E**) Example of representative flow cytometry data showing the in vitro reactivity of TCR-transduced T cells expressing TR-TCRs or NTR-TCRs in the presence of MC38 tumor cells. Shown are data for the TCRs derived from the following clonotypes, as listed in data S15: CT17.53, CT16.26, and CT17.8. (**F**) Overview of the antitumor reactivity of the 48 CD8+ TCRs selected on the basis of the gene signature. TCRs are grouped on the basis of predicted reactivity.

**Fig. 9.**
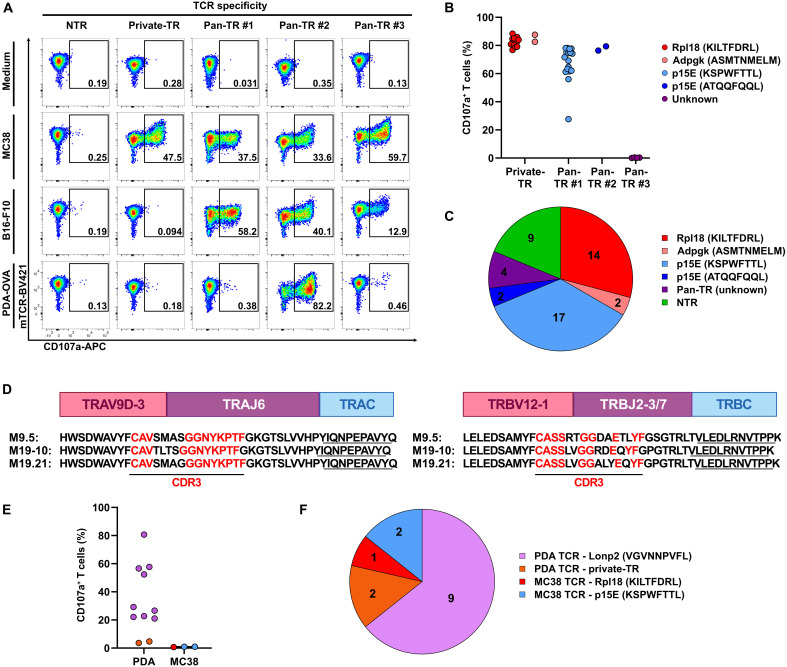
Differentiation of MC38-derived CD8+ TR-TCRs based on tumor reactivity and antigen specificity. (**A**) Example of representative flow cytometry data showing the in vitro reactivity of TCR-transduced T cells expressing TR-TCRs or NTR-TCRs in the presence of the indicated tumor cells. Shown are data for the TCRs derived from the following clonotypes, as listed in data S15: CT17.26, CT17.1, CT17.20, CT15.8, and CT15.36. (**B**) Overview of the antigen-specific reactivity of the 39 CD8+ TR-TCRs grouped on the basis of their tumor-reactivity pattern. See fig. S12A for representative flow cytometry data. (**C**) Pie chart summarizing the tumor- and antigen-specific reactivity of all 48 MC38-derived TCRs tested. (**D**) Display of the similarity of the variable and CDR3 domains of the TCRα/β pairs of the three LONP2-reactive TCRs as identified in the PDA model. (**E**) Overview of the reactivity of the 14 TCRs selected on the basis of sequence homology against the LONP2/VGVNNPVFL epitope (data S16), grouped on the basis of the tumor of origin. See fig. S12B for representative flow cytometry data. (**F**) Pie chart summarizing the tumor- and antigen-specific reactivity of the 14 TCRs tested.

The reactivity of T cells expressing p15E/ATQQFQQL– and LONP2/VGVNNPVFL–specific TCRs against other tumor cells of C57BL/6 mouse origin (Fig. 6, A and B, and fig. S7, A and B) raised the question of whether TCR clonotypes with these antigen specificities could also be identified in the tumor-infiltrating T cell repertoire of these tumors. We therefore performed scRNA-seq on CD3+ T cells isolated from MC38 tumors and evaluated the CD8+ TCR repertoire by means of the workflow as described above. The resulting UMAP T cell landscape showed prominent T_ex_ and T_pex_ clusters, and application of the TR-95 and NTR-57 gene signatures, as defined on the basis of our PDA tumor model, implied the presence of both TR and bystander T cells in MC38 tumors (Fig. 8, A to C, and fig. S11, A to D). As before, we determined the TR and NTR scores for each of the expanded TCR clonotypes by calculating the mean scores of the T cells included. The result was in line with that of the PDA model, in that it revealed a segregation of clonotypes predicted as either TR or bystander T cells (Fig. 8D). From this repertoire, we sampled a total of 48 clonotypes, of which 39 were predicted to be TR and 9 NTR. The outcome of in vitro functional testing of these TCRs against MC38 cells was in full correspondence with this prediction (Fig. 8, D to F). Furthermore, testing against B16 and PDA tumor cells revealed four reactivity patterns, featuring reactivity against MC38 only, as well as different degrees of reactivity against B16 and PDA tumor cells (Fig. 9A).

Three independent reports have described a total of 11 different mutanome-encoded neoepitopes in the MC38 model (*62*–*64*). Screening of the 16 TCRs uniquely reactive against MC38 showed that all were specific for either of the following two neoepitopes: Rpl18/KILTFDRL or Adpkg/ASMTNMELM (Fig. 9, B and C, and fig. S12A). Accordingly, these were reported as the immunodominant neoepitopes in, respectively, mice immunized with irradiated MC38 tumor cells (*62*, *63*) and mice rejecting MC38 tumors upon ICB treatment (*63*). All TR-TCRs were furthermore screened against the retroviral p15E/KSPWFTTL epitope, which has also been reported as an immunodominant antigen in MC38 (*45*, *47*, *65*), as well as the p15E/ATQQFQQL and LONP2/VGVNNPVFL epitopes for which we identified specific TCRs in our PDA model. All 17 TCRs that mediated strong T cell reactivity against MC38 and B16 were found to react against the p15E/KSPWFTTL epitope. This is concordant with published data by others showing that this is a highly immunogenic epitope in both M38 and B16 (*45*, *65*), as well as our own data showing that this epitope is not expressed in the PDA tumors (Fig. 6F). The two TCRs mediating strong T cell reactivity against MC38, B16, and PDA tumor cells were found to react against the p15E/ATQQFQQL epitope, in line with our finding that TCRs targeting this epitope as isolated from our PDA tumor model also mediate reactivity against MC38 and B16 tumor cells (Fig. 9, B and C, and fig. S12A). The further four TCRs that did not react against the p15E peptides were neither reactive against the LONP2/VGVNNPVFL epitope nor the Cripto-1/SAFEFGPVA epitope that was reported by others (*46*, *47*), indicating that these TCRs must target one or more as yet unidentified antigens.

In the context of our search for LONP2/VGVNNPVFL–reactive TCRs in the MC38 model, we noticed that the three TCRs targeting this epitope as identified in the PDA model showed a notable conservation in their sequences for both the α and β chains (Fig. 9D). On the basis of this, we readily identified 11 further highly similar TCRα/β pairs in the PDA dataset but could only find three moderately similar TCRs in the MC38 model. Functional testing of these TCRs, all of which were TR, reflected this observation, in that the majority (9 of 11) of the PDA tumor–derived TCRs, but none of the three MC38-derived TCRs, mediated T cell reactivity against the LONP2/VGVNNPVFL peptide. Instead, the latter TCRs were found to react against one of the immunodominant epitopes in MC38, either p15E/KSPWFTTL or Rpl18/KILTFDRL (Fig. 9, E and F, and fig. S12B).

Together, our survey of the TR-TCR repertoire in MC38 tumors, as summarized in Fig. 9C, showed that our TCR screening pipeline is highly robust and furthermore confirmed that the p15E/ATQQFQQL epitope is a target of the natural T cell response both in our PDA model and in MC38 tumors. Notably, the latter was already reported for B16 tumors (*59*, *60*) but not for MC38 (*45*, *65*), likely due to MC38 expressing at least three other immunodominant epitopes. This also offers a plausible explanation for the lack of LONP2/VGVNNPVFL–reactive TCRs in our MC38 dataset and is therefore not in contradiction with our finding that MC38 tumor cells are recognized by LONP2/VGVNNPVFL–reactive TCRs as isolated from the PDA tumors.

## DISCUSSION

We recently performed a detailed analysis of the tumor-infiltrating T cell response in human pancreatic cancer at the single-cell level, resulting in the identification of a diversity of TR-TCR clonotypes, thereby providing a perspective for personalized T cell therapy in this treatment-resistant indication (*14*). In the present study, we conducted a similar analysis in an orthotopic mouse model for pancreatic cancer toward preclinical evaluation of this therapeutic concept. The resulting dataset comprises scRNA-seq data of >54,000 tumor-infiltrating T cells and functional TCR testing of 129 TCR clonotypes. Also in mouse pancreatic tumors, we found many TCRs that mediated tumor recognition. Investigation of the antigen specificity of the CD8+ T cell response revealed three epitopes that represent distinct, clinically relevant classes of tumor antigens: the HOOK3/MHMQNTVSL neoepitope encoded by a somatic mutation in the *Hook3* gene, the p15E/ATQQFQQL epitope derived from the ectopically expressed p15E retroviral antigen, and the LONP2/VGVNNPVFL epitope derived from the cell stress–induced LONP2 autoantigen. Whereas the neoepitope is a “private” antigen, only expressed by the pancreatic tumor model subject of our study, the other two antigens are expressed more broadly, including by the independently derived MC38 and B16 tumor models that represent different tumor types.

In view of the latter, we subsequently also dissected the tumor-infiltrating T cell repertoire in MC38 tumors. In accordance with prior studies by others (*45*, *62*–*65*), we found the TR T cell repertoire to primarily respond to three immunodominant epitopes, the retroviral p15E/KSPWFTTL epitope and the mutanome-encoded neoepitopes Rpl18/KILTFDRL and Adpkg/ASMTNMELM. In addition, we found TCRs reacting against the p15E/ATQQFQQL epitope that is shared by the MC38, B16, and our PDA tumor models. On the basis of a conserved motif in the TCRα/β variable sequences of the three identified LONP2/VGVNNPVFL–reactive TCRs, we could readily identify further TCRs with this reactivity in the PDA dataset but not in the MC38 dataset. Most likely, this is due to the expression of the aforementioned immunodominant epitopes in the MC38 model. The establishment of an antigen immunodominance hierarchy is a multifactorial process influenced by quantitative and qualitative differences between antigens/epitopes related to antigen processing and presentation, as well as by the available T cell repertoire (*22*, *66*–*68*). By way of comparison, TCR clonotypes directed against the immunodominant OVA/SIINFEKL epitope were readily identified in the scRNA-seq datasets of all PDA-OVA tumors, whereas only 1 of the 26 OVA-reactive TCRs tested was directed against the subdominant OVA/KVVRFDKL epitope.

Considering all 148 CD8+ TCRs tested (86 from the PDA tumors, 48 from MC38, and 14 from either model based on the conserved motif identified in the LONP2/VGVNNPVFL–reactive TCRs), our TCR discovery pipeline involving gene signature–based prediction and in vitro testing of TCR reactivity is robust, in that TR-TCR clonotypes were identified with a true positive rate of 100% (111 of 111). Testing of the 37 expanded TCR clonotypes displaying an NTR gene signature revealed that, accordingly, 33 (90%) did not mediate reactivity against the corresponding tumor cells in vitro, suggesting that these represent bystander T cells activated by microbial exposure (*48*, *49*). Whereas, for four of these TCRs, tumor reactivity was observed, this is most likely due to the enhanced antigen presentation in the in vitro setting. Notably, the primary aim of the present study was the charting of the tumor-infiltrating TCR repertoire that mediates T cell reactivity against the tumor cells in vivo, as well as to evaluate their antitumor reactivity pattern and antigen specificity.

As for the LONP2-reactive T cells identified, Lon proteases play an important role in protecting cells against oxidative stress by degrading proteins damaged by reactive oxygen species (*61*). Whereas the proteasome is responsible for degrading damaged proteins in the cytoplasm, nucleus, and endoplasmic reticulum, the LONP1 and LONP2 isoforms mediate protein homeostasis in the mitochondria and peroxisomes, respectively. The enzymes are evolutionary highly conserved, illustrating their importance for cellular integrity. Given the notion that tumor growth is associated with oxidative stress, it is expected that both LONP1 and LONP2 have been found expressed at increased levels in multiple human cancers and that gene silencing suppresses tumor growth (*61*). This implies that conditions of cellular stress can also lead to enhanced LONP expression in nontransformed cells. On the one hand, this readily explains why we found T cells expressing LONP2-specific TCRs to react against normal mouse splenocytes, because under standard in vitro culture conditions, these are exposed to oxidative stress (*69*). On the other hand, this suggests that normal somatic tissues exposed to stress may also be targeted by such T cells, for instance, in patients with cancer who are subjected to cytostatic treatments (*70*). Whether or not this would bring harm to the patient is likely to depend on the magnitude of the T cell response, as can be illustrated on the basis of extensive clinical knowledge concerning the therapeutic index of the T cell response against the melanoma-associated MART-1 antigen, in particular the HLA-A*0201–restricted MART-1_26–35_ epitope. Whereas spontaneous and vaccine-induced T cell responses against this epitope may be associated with varying degrees of autoimmune skin depigmentation without severe clinical complications, the infusion of patients who have melanoma with autologous T cells engineered to express a TCR directed against this epitope was associated with a high risk for dose-limiting on-target toxicity involving T cell–mediated destruction of normal melanocytes in the skin, the eye, and the inner ear (*71*).

In view of the above, epitopes encoded by ectopically expressed endogenous retroviral sequences, as exemplified by the p15E epitope, may offer safer targets for adoptive T cell therapy. The human genome also harbors endogenous retroviral sequences [human endogenous retroviruses (HERVs)] that, while typically silenced in healthy somatic tissues, were found ectopically expressed in a variety of human cancers. T cell responses against HERV-encoded antigens have been detected in patients with cancer, and these responses were proposed to be involved in ICB-induced antitumor immunity (*72*). Evidence from preclinical models suggests that epigenetic therapy by means of DNA methyltransferase inhibitors and/or histone deacetylase inhibitors may synergize with ICB through the induction of HERV-antigen expression in tumors (*73*, *74*).

A limitation of our study is that the aforementioned epitopes reflect the specificity of only 15 of the 60 TR-TCR clonotypes identified in our PDA model that mediate reactivity through non-OVA T cell epitopes. This suggests that neither of the three antigens targeted by these clonotypes are immunodominant. In the case of a highly immunogenic antigen such as OVA/SIINFEKL, this hierarchy is very prominent, but in the case of two or more sub/codominant antigens, the outcome may be variable and therefore differ between individual mice challenged with the same tumor (*66*). On the basis of MHC restriction and reactivity against different C57BL/6 tumor cell lines, the TR-TCR repertoire in the PDA model is expected to target at least one further D^b^-restricted and one further K^b^-restricted “private” neoepitope, as well as several recurrent MHC class I– and class II–restricted tumor antigens. Although we screened all “private” TR-TCRs against our panel of candidate neoepitopes, this did not result in further hits. Epitope prediction based on prediction of MHC binding is inaccurate (*75*), a main reason being that the naturally processed endogenous epitope repertoire is governed by multiple restrictions in addition to epitope-MHC binding (*76*). Furthermore, there is increasing evidence that tumor-associated epitopes include nonannotated peptide sequences that are encoded by noncanonical transcripts or arise from posttranslational events (*77*), most recently also from studies in human PDAC (*78*). Last, the hit rate of immunopeptidomics screens is limited by technical aspects (*79*, *80*), a notorious issue being the loss of hydrophobic peptides resulting from adsorption to laboratory materials such as tubes and pipette tips (*81*). This is likely to hamper the detection of, in particular, K^b^-restricted peptides because of the fact that the corresponding sequence motif primarily features amino acids with hydrophobic side chains, in particular leucine, isoleucine, valine, tyrosine, phenylalanine, and alanine (*51*). The fact that the H-2K^b^–restricted p15E/ATQQFQQL epitope comprises four polar glutamine (Q) residues may have facilitated its detection in our peptidome analyses. A second technical aspect may be the limited quantity of mutanome-encoded neoepitopes as compared to tumor-associated self-antigens, as noted by others in the field (*79*, *80*). This could explain why we identified the private D^b^-restricted Hook3 epitope by means of reverse immunology but could not detect it in our immunopeptidome fractions.

In summary, our proof-of-concept study shows that, by means of our TCR discovery pipeline, TR-TCR clonotypes can readily be identified on the basis of their transcriptional state in scRNA-seq datasets from TILs in mouse tumor models, in a manner analogous to what we reported for human PDAC tumor samples (*14*, *19*). Whereas the current focus of the field is on mutanome-encoded neoepitopes (*9*, *50*, *82*), the natural antitumor T cell response in mouse PDAC tumors appears to be primarily directed against recurrent tumor antigens. Also, our analyses of the natural TIL repertoire in human PDAC tumors thus far did not reveal reactivity against predicted mutanome-encoded neoepitopes (*14*). While further experiments in the mouse model will provide insight into the therapeutic potential and safety of these two antigen classes with regard to personalized T cell therapy, our primary objective is now to shed more light on the nature and origin of the epitopes that are targeted by the natural TIL repertoire in human PDAC tumors.

## MATERIALS AND METHODS

### Cell lines and culture

PDA30364 (hereafter referred to as PDA) and PDA30362 tumor cell lines were generated from primary pancreatic tumors in the Elas-tTA/TetO-Cre Kras+/LSL-G12D Trp53+/LSL-R172H mouse line. The PDA-OVA tumor line was derived by lentiviral transduction with the pLenti6.3_3xFLAG-Ovalbumin-F2A-EGFP construct (Bayer Pharma AG), as described previously (*17*). From this cell line, several clonal lines were derived, one of which (PDA-OVA#7) upon injection into immunocompetent C57BL/6 mice led to the outgrowth of a tumor in a single mouse with ID no. m14865. From this tumor, a cell line (PDA-OVA#7_m14865; here referred to as PDA-OVA) was established that expressed lower OVA levels than its predecessor and grew out in most challenged mice (see fig. S1). CRISPR-Cas9 was used to generate PDA knockout variants lacking MHC class I with single guide RNAs targeting β2-microglobulin or H-2K^b^/H-2D^b^ for selective knockouts and l-A^b^ for MHC class II knockout. Murine melanoma cell lines B16-F10, B cell lymphoma line B771, and murine colon carcinoma cell lines MC38 (MC38-L) (*62*, *63*) originated from Leiden University Medical Center, The Netherlands. B cell lymphoma line B771 (*83*) was modified with transposon pSB_tet_RB_iOVA to express chicken OVA. T2_K^b^ and T2_D^b^ cells were provided by T. van Hall (Leiden University Medical Center). PDA cells were cultured in Dulbecco’s modified Eagle’s medium (DMEM) supplemented with 1 mM sodium pyruvate; PDA-OVA cells are selected with blasticidin (10 μg ml^−1^). B16-F10 cells were cultured in RPMI supplemented with 1 mM sodium pyruvate. MC38 cells were cultured in Iscove’s modified Dulbecco’s medium supplemented with 50 μM β-mercaptoethanol. T2 cells and B771/-OVA cells were cultured in Iscove’s modified Dulbecco’s medium supplemented with 2 mM l-glutamine. All cell culture media were supplemented with 10% fetal bovine serum, penicillin (100 units ml^−1^), and streptomycin (100 μg ml^−1^). For the in vitro TCR-screening assay, we made use of a human T cell line (T222) that originated from an ex-vivo–expanded culture of tumor-infiltrating T cells isolated from a human PDAC sample (PDA222), as described previously (*11*, *19*). T cells were expanded by a rapid expansion protocol (*84*) to high numbers and frozen in aliquots. Enrichment for either CD8+ or CD4+ T cells before expansion resulted in T cell batches in which most T cells were respectively CD8+ or CD4+. Before use in TCR screening assays, the CD4/CD8 ratio was evaluated by flow cytometry. Batches comprising at least 80% of either CD8+ or CD4+ T cells were used for the analysis of TCRs derived from, respectively, CD8+ and CD4+ TCR clonotypes (*19*). All cell lines were routinely screened for mycoplasma contamination by polymerase chain reaction.

### Mice and in vivo experiments

Growth of PDA-OVA (no. 7) tumor cells was analyzed in NOD.Cg-Prkdcscid Il2rgtm1Wjl/SzJ and C57BL/6 wild-type mice by injecting 2 × 10^6^ or 5 × 10^6^ PDA-OVA (no. 7) cells, respectively, subcutaneously into the flank in phosphate-buffered saline (PBS)/Matrigel (1:1, Corning). Tumor growth was measured twice a week with a caliper. The tumor volume was calculated by multiplying length by width by height. For assessing the impact of anti–PD-L1, treatment was initiated 1 week after tumor implant when tumors were palpable and started to grow out. For ICB treatment, 200 μg of PD-L1 [Atezolizumab, Selleckchem, no. A2004, human immunoglobulin G1 (IgG1)] was administered intraperitoneally. As isotype control, mouse IgG1 control (BioXCell, clone MOPC-21, BP0083) was used. Mice were euthanized if signs of distress were noticed, when termination criteria were reached, or when analyses were performed at specific time points. For the generation of tumor material for single-cell sequencing of TILs, male C57BL/6-Ly5.1 mice at the age of 6 to 12 weeks were injected orthotopically with 10 μl of PBS:Matrigel containing 1 × 10^6^ PDA30364/-OVA cells into the pancreas. Once tumors were palpable after 2 to 3 weeks, mice were taken down for analysis, unless termination criteria due to tumor size were met beforehand. Tumors were harvested, minced into smaller pieces with a scalpel, and digested using the human tumor dissociation kit with a gentleMACS Octo tissue dissociator (Miltenyi) according to the manufacturer’s instructions (program “37C_h_TDK_3”). Following 1-hour digestion, tumor cell suspensions were poured through a 100-μm strainer precoated with 2.5% bovine serum albumin/PBS. Next, cells were washed twice over a 70-μm cell strainer and frozen in liquid nitrogen in freezing media (fetal bovine serum + 10% dimethyl sulfoxide) until processing for scRNA-seq. The presence of OVA/SIINFEKL–reactive T cells in dissociated PDA/PDA-OVA tumors was detected with OVA/SIINFEKL H-2K^b^-APC (allophycocyanin) dextramers (Immudex, no. JD2163). A dextramer containing ADPGK, a C57BL/6-derived neoepitope, was used as a background control. Mice were bred in the animal facilities at the German Cancer Research Center. All animal procedures followed the institutional laboratory animal research guidelines and were approved by the governmental authorities (Regional Administrative Authority Karlsruhe, Germany; study approval numbers A-12/18, G-5/15, G-222/15, and G-271/15).

### scRNA-seq and V(D)J receptor sequencing

For sorting of cells, dissociated tumors were thawed into preheated bovine serum albumin/PBS (2.5%) at 37°C. Dead cell staining, unspecific Fc blocking, and Ab staining were performed as described before (*17*). The following Abs against mouse were used for extracellular staining: CD45-PE/Dazzle594 (BioLegend, 1:1000, clone 30-F11, no. 103145), CD3-FITC (fluorescein isothiocyanate) (BioLegend, 1:200, clone 17A2, no. 100204), CD90.2-AF700 (BioLegend, 1:200, clone 20-H12, no. 105320), CD8a-APC/Cy7 (BioLegend, 1:200, clone 53-6.7, no. 100714), CD4-BV605 (BioLegend, 1:200, clone RM4-5, no. 100548), CD279-PE/Cy7 (BioLegend, 1:200, clone 29F.1A12, no. 135216), and CD11b-PerCP/Cy5.5 (BioLegend, 1:1000, clone M1/70, no. 101228) to sort on live CD45+/Cd11b−/CD90.2+/CD3+ T cells using BD FACSAria Fusion at Core Facility Flow Cytometry at German Cancer Research Center Heidelberg. Approximately 18,000 cells were loaded as input for scRNA-seq and V(D)J TCR sequencing, which was done with 10x Genomics v1 chemistry according to the manufacturer’s protocol (no. CG000086 Rev K). Sequencing of libraries was done at Genomics and Proteomics Core Facility at German Cancer Research Center Heidelberg using a NovaSeq 6000 (Illumina) with a target read depth of 20,000 per cell for the gene expression library and 5000 for the V(D)J library. Sequencing data were aligned using CellRanger (version 3.1), using mouse reference genome mm10-3.0.0 for the gene expression library and vdj_GRCm38_alts_ensembl-3.1.0 for the V(D)J library. Subsequent data analysis of output FeatureBarcodeMatrix was done in R (version 4.0.0) using the Seurat (version 4.0.3) data analysis workflow. Briefly, data of individual PDA or PDA-OVA mice were preprocessed independently for quality metrics including the percentage of mitochondrial genes, unique molecular identifiers per cell, and genes detected per cell. Using clonotype information from V(D)J library sequencing, TCR hypervariable region CDR3 information of T cells was added into metadata on the basis of barcodes. Next, individual Seurat objects were merged into single objects each for PDA or PDA-OVA models and processed using NormalizeData, FindVariableFeatures, and ScaleData functions. Following dimension reduction with RunPCA, data were integrated using Harmony (version 1.0). Analysis of initial clusters revealed small clusters other than T cells expressing markers of monocytes, NK (natural killer) cells, B cells, and cancer-associated fibroblasts, which were removed from the dataset. To display all T cell clusters, data was reprocessed as described above, but to avoid bias from expanded T cell clonotypes, TCR-associated genes were not considered as variable features during principal components analysis. To zoom into CD8 or CD4 T cells, we used a multistep involving cluster-based subsetting as a first step. Second, CD8 T cells marked by the expression of *Cd8a* and/or *Cdb8b1* > 0 with expression of *Cd4* or *Foxp3* == 0 were recovered from the CD4 Seurat object and merged back into the CD8 T cell object. Next, any CD4 T cells that were still in CD8 T cell clusters were moved into CD4 T cell clusters on the basis of their feature expression of *Cd4* > 0 & *Cd8a* == 0 & *Cd8b1* == 0 or cells expressing *Foxp3* > 0 & *Cd8a* == 0 & *Cd8b1* == 0. Last, CD8 and CD4 T cell subsets were reprocessed using the Seurat pipeline and analyzed further. The number of principal components was determined by ElbowPlot, and desired cluster granularity was determined on the basis of genes identifying biological subtypes of CD8 or CD4 T cells. FindAllMarkers was used to identify differentially expressed genes between clusters. For this, the default Wilcoxon rank-sum test was used, but an alternative calculation using logistic regression was largely similar. Clonality was investigated using Immunarch (version 0.6.7) using repClonality method “rare.” The AddModuleScore function from Seurat was used to calculate scores of various gene signatures. Thresholds in “q99” as max.cutoff and quantile “q1” as min.cutoff were applied to remove outlier bias in FeaturePlot. Nebulosa (version 1.0.1) was used for displaying density plots, and the viridis (version 0.6.2) color package was used for visualization. To determine the predictive value and fit of different signatures with the outcome of tested TR/NTR-TCRs, ROC was performed using ROCit (version 2.1.1), and pROC (version 1.18.0) was used for identification of AUC confidence intervals. Other packages used for visualization and miscellaneous operations are ggplot (version 3.3.5), ggrepel (version 0.9.1), patchwork (version 1.1.1), tibble (version 3.1.0), dplyr (version 1.0.5), Hmisc (version 4.5.0), and stats (version 4.0.0).

For the analysis of the MC38 tumors, procedures were essentially the same as described above, except for the following details. Single-cell RNA and V(D)J TCR libraries were generated using 10x Genomics version 2 chemistry according to the manufacturer’s protocol (no. CG000331 Rev E). Sequencing data were aligned using CellRanger (version 8.0.1), using mouse reference genome mm10-2020-A for gene expression libraries and vdj_GRCm38_alts_ensembl-5.0.0 for V(D)J libraries. Subsequent data analysis of output FeatureBarcodeMatrix was done in R (version 4.3.3) using the Seurat (version 4.3.0) data analysis workflow, as described above.

### Immunochemistry

Immunohistochemistry of PDA-OVA and PDA tumor samples for infiltration of CD3+ T cells was performed as described previously using rabbit monoclonal anti-mouse CD3 (1:200, Abcam, clone SP7, no. ab16669) (*17*). Staining for MHC class I was performed on frozen tumor sections. Staining was done by D. Heide from the group of M. Heikenwälder at the German Cancer Research Center Heidelberg using anti–MHC-I Ab (Abcam, no. ab15681, clone ER-HR 52, 1:500 dilution) as well as the Bond Polymer Refine Detection kit (Leica Biosystems) and counterstained using hematoxylin. Cytokeratin-19 staining was performed on serial sections using anti-CK19 Ab (DSHB, no. TROMA-III-s, 1:100 dilution) as well as the DAB Substrate Kit with horseradish peroxidase (brownVector Laboratories). The sections were counterstained using Alcian blue.

### Expression of murine TCRs in human effector cells

Matched full-length TCR α- and β-chain nucleotide information on single-cell levels was obtained from V(D)J receptor sequencing. Clonally expanded and predicted TCR clonotypes were synthesized as DNA oligomers by Twist Bioscience. Golden Gate assembly was used for seamless cloning of TCR α and β chains as a single open-reading frame separated by a self-cleaving P2A site into the pcDNA3.1 plasmid. Similarly, vectors containing a full-length murine CD8α, CD8β, or CD4 co-receptor were generated. The T7 mScript Standard mRNA Production System (Biozym, no. 150352) was used on Not I–linearized DNA plasmids containing TCR/co-receptor information for the generation of capped and tailed mRNA. mRNA quality was routinely checked as a single peak on the 2100 Bioanalyzer system using Agilent RNA 6000 Nano chips. mRNA was transfected into human T cells using the Gene Pulser Xcell electroporation system (Bio-Rad) using a 4-mm cuvette and single square-wave pulse for 5 ms at 500 V at a ratio of 2 μg mRNA encoding murine TCR and 2 μg murine co-receptor each per 1 × 10^6^ cells. Before electroporation, T cells were rested for 3 days after thawing with interleukin-2 (300 IU/ml). Twenty-four hours after electroporation, T cells expressing murine TCR and co-receptor were used for T cell activation assays. For further information, see our previously published work (*14*, *19*).

### T cell activation assay

T cell activation was analyzed in a 5-hour coculture assay of T cells expressing murine TCRs and co-receptors with various target conditions to determine tumor and target specificity. Readout for specific T cell activation was assessed with staining of intracellular levels of TNFα or cell surface expression of CD107a by flow cytometry. As target conditions, tumor cell lines or tumor digests were pretreated or used as is with T cells at an effector-to-target ratio of 200,000 T cells to 50,000 tumor cells in a 96-well U-bottom plate format. Where the degranulation marker CD107a was investigated, anti-human CD107a-APC (BioLegend, 1:40, clone H4A3, no. 328620) was added directly to the coculture. One hour into the coculture, BD GolgiPlug (Brefeldin A) and BD GolgiStop (Monensin) were added at 1:1000 each to inhibit intracellular protein transport processes, resulting in the accumulation of TNFα cytokine in response to T cell activation inside the cell and allowing for detection of the degranulation marker CD107a, respectively. After a total of 5 hours, cells were processed for extracellular and intracellular staining. Plates were centrifuged at 400*g* for 4 min. Cells were washed twice with PBS, followed by dead cell discrimination with LIVE/DEAD Fixable Aqua (Thermo Fisher Scientific, 1:400, no. L34957) or Zombie Violet Fixable Viability Kit (BioLegend, no. 423114) for 15 min at 4°C. Unspecific binding of Abs was reduced by incubation with Fc blocking Abs against CD16/32 (BioLegend, 1:100, clone 93, no. 101302) and CD16.2 (FCyRIV, BioLegend, 1:50, clone 9E9, no. 149502) for an additional 10 min at 4°C. Cells were washed twice with flow buffer (PBS) before performing extracellular staining with the following anti-human Abs for 30 min at 4°C: CD3-BV711 (BioLegend, 1:40, clone OKT-3, no. 317328), CD4-APC-Fire 750 (BioLegend, 1:200, clone SK3, no. 344638), and CD8-AF700 (BioLegend, 1:40, clone Hit8a, no. 300920). Transfection efficiency in human T cells was checked using anti-mouse Abs for TCR β-chain-BV421 (BioLegend, 1:20, clone H57-597, no. 109230), and in the case of CD8 TCRs, murine CD8α and CD8β co-receptors, using CD8a-PerCP (BioLegend, 1:100, clone 53-6.7, no. 100732) and CD8b-PE (BioLegend, 1:100, clone YTS156.7.7, no. 126608), or in the case of CD4 TCRs, murine CD4 co-receptor, using CD4-PE (BioLegend, 1:100, clone GK1.5, no. 100408). Cells were washed twice with flow buffer before fixation and permeabilization with BD Cytofix/Cytoperm Fixation/Permeabilization Solution (Thermo Fisher Scientific, no. BDB554715) according to the manufacturer’s protocol. Intracellular staining against human TNF-a-AF488 (BioLegend, 1:20, Mab11, no. 502915) was done for 30 min at 4°C. Cells were washed twice using flow buffer and analyzed with a BD LRS Fortessa flow cytometer. Data were analyzed with FlowJo (version 10.8.1, FlowJo LLC). As a positive control for T cell stimulation, phorbol 12-myristate 13-acetate (100 ng ml^−1^) and ionomycin (500 ng ml^−1^) were used for unspecific activation of T cells. For induction of MHC class I on PDA and PDA-OVA, tumor cell lines were pretreated with recombinant murine IFN-γ (300 ng ml^−1^; ImmunoTools) for 2 days. When PDA cells were also pretreated with 100 nM decitabine (5-aza-2′deoxycytidine), media containing drug were replaced every 24 hours. Ex vivo tumor digests of PDA/PDA-OVA were pretreated with IFN-γ (300 ng ml^−1^) for 24 hours to increase expression levels of MHC class I and class II. For induction of MHC class II on PDA and PDA-OVA, tumor cell lines were treated with a combination of IFN-γ (300 ng ml^−1^) and 100 nM MEKi GDC-0623 for 2 days. Surface expression was monitored using following Abs against mouse: I-A/I-E-APC/Cy7 (BioLegend, 1:100, clone M5/114.15.2, no. 107627), H-2Kb-APC (BioLegend, 1:100, clone AF6-88.5, no. 116518), H-2Db-FITC (BioLegend, 1:100, clone KH95, no. 111506), and H-2K^b^ bound to SIINFEKL-PE (BioLegend, 1:100, clone 25-D1.16, no. 141604). To determine epitope specificity, synthetic peptides were loaded onto cells by adding peptide (1 to 10 μg ml^−1^) for 1 hour at 37°C to presenting cells. Unbound peptides were subsequently washed out before using peptide-loaded cells in a coculture assay. Peptides eluted by mild acid elution were similarly loaded onto T2_K^b^ or T2_D^b^ cells for testing of specific detection of naturally processed and presented peptide recognition by TCR-transfected T cells in coculture assay. A TCR was considered reactive if the change in TNFα levels upon coculture of TCR-transfected T cells with a target condition was higher than at least >0.3% after subtracting 2 standard deviations of TNFα levels of respective TCR-transfected T cells against unstimulated control (medium). The strength of reactivity was subclassified on the basis of following TNFα thresholds into weak TCRs ∆ > 0.3%, intermediate TCRs ∆ > 1.5%, and strong TCRs ∆ > 5%. Values in graphs represent mean TNFα values from at least two biological replicates.

### Expression and functional testing of murine TCRs in primary mouse T cells

Phoenix-Ampho packaging cells were maintained in DMEM supplemented with 10% heat-inactivated fetal bovine serum, 1% penicillin-streptomycin, 1% l-glutamine, and 25 mM Hepes (complete DMEM) at 37°C and 5% CO_2_. To produce amphotropic murine leukemia virus–pseudotyped retroviruses, Phoenix-Ampho cells were seeded 1 day before transfection on tissue-cultured plates precoated with poly-l-lysine to promote adherence and uniform growth, aiming for ~70% confluence at the time of transfection. Thirty minutes before transfection, culture medium was replaced with complete DMEM lacking penicillin-streptomycin. Cells were then transiently transfected with pMX retroviral vectors encoding the TCR α- and β-chain sequences (*85*) using Lipofectamine 2000 (Thermo Fisher Scientific) according to the manufacturer’s instructions. The medium was replaced with fresh complete DMEM lacking penicillin-streptomycin 6 to 8 hours after transfection, and viral supernatants were collected at 48 hours posttransfection, filtered (0.45 μm), and used immediately for T cell transduction.

Toward TCR gene transfer into primary mouse T cells, spleens were collected from P14 × CreCD4 transgenic mice, and single-cell suspensions were prepared by mechanical disruption through a 40-μm cell strainer. Erythrocytes were lysed using ACK lysis buffer (Gibco, Thermo Fisher Scientific). Splenocytes were cultured in complete RPMI 1640 medium supplemented with 10% fetal bovine serum, 1% penicillin-streptomycin, 2 mM l-glutamine, 20 mM Hepes, 1 mM sodium pyruvate, 100 μM nonessential amino acids, and 50 μM β-mercaptoethanol. Cytokines were added at final concentrations of IL-2 (129 IU/ml), IL-7 (2.5 pg/ml), and IL-15 (2.5 pg/ml), together with concanavalin A (2 μg/ml; Sigma-Aldrich). Cells were seeded at a density of 3 × 10^6^ cells/ml and incubated overnight at 37°C in 5% CO_2_. The following day, 5 × 10^6^ cells were transduced by spinoculation with amphotropic retroviral supernatants derived from pMX-based vectors encoding the indicated TCR α/β-chain sequences. Transductions were performed in six-well, non–tissue culture–treated plates precoated with RetroNectin (25 μg/ml; Takara Bio) by centrifugation at 3200*g* for 120 min at 4°C. A second round of spinoculation was carried out 24 hours later using 2.5 ml of freshly collected viral supernatant. On the following day, transduced cells were cocultured with irradiated target tumor cells for 3 days in complete RPMI medium supplemented with IL-2, IL-7, and IL-15 to support activation before use in downstream functional assays. Functional assays were performed as described above using the following two Abs: anti-mouse CD3-FITC (100204, BioLegend) and anti-mouse CD107a-APC (121614, BioLegend).

### Synthetic peptides and peptide libraries

Synthetic peptides were chemically synthesized and purified by the group of S. Eichmueller at the German Cancer Research Center Heidelberg. Details of synthesis and purification are as described previously (*17*). Synthetic H-2K^b^ and H-2D^b^ peptide libraries were provided by S. Stevanovic (University of Tübingen). The H-2K^b^ library consisted of ~10^6^ peptides with the sequence xxxxFxxI/L/M. The H-2D^b^ library contains around 10^9^ peptides with the sequence xxxxNxxxI/L/M, where x indicates a random position for all 20 amino acids.

### Mild acid elution of naturally processed and presented peptides from MHC

Mild acidic elution (MAE) from ~100 × 10^6^ cells was performed according to the protocol described by Sturm *et al.* (*53*) and cited publications therein. Briefly, cells that were pretreated for 2 days with IFN-γ were treated daily as follows for 5 consecutive days: The medium was removed from cells in a flask and then washed twice with 8 ml of Hanks’ balanced salt solution (+Mg, +Ca; Gibco, no. 14025092) to remove residual tissue culture medium. Each flask was treated for 90 s at room temperature with an appropriate minimum volume to cover the cells (2.5 ml per flask) of citric acid buffer (0.067 M citric acid and 0.123 M Na_2_HPO_4_, adjusted with NaOH to pH 3.1), to denature surface MHC molecules, and to release MHC-associated peptides into the supernatant. The obtained peptides were desalted by reverse-phase purification using a SepPak 96-well plate (Waters, no. 186003966). Peptides eluted in 28% acetonitrile (ACN) in 0.1% trifluoroacetic acid (TFA) were dried by vacuum centrifugation (Concentrator Plus, Eppendorf).

### LC-MS of the MAE sample

Dried eluate harvested via MAE from 1 day was dissolved in 15 μl of 5% ACN in 0.1% TFA containing a matrix [Peptide Retention Time Calibration (PRTC) Mixture (25 fmol/μl; Pierce, no. 88321) and bovine serum albumin digest (75 fmol/μl; Pierce, no. 88341)] followed by 3-min bath sonication. Only 5 μl was taken for analysis as two technical replicates by liquid chromatography (LC; U-3000, Thermo Fisher Scientific) coupled to an Orbitrap Exploris 480 (Thermo Fisher Scientific). The above matrix (free of target peptides) was systematically injected before the analysis of the MAE sample as a negative control to test for the absence of any artifact, e.g., because of possible carryover of peptides from previous analyses. The LC gradient based on solvent A [0.1% formic acid (FA) in H_2_O] and solvent B (100% ACN and 0.1% FA) consisted of multiple segments as follows: 2 to 6% B (98 to 96% A) for 5 min, 6 to 28.5% B for 75 min, 28.5 to 48% B for 27 min, 48 to 80% for 2 min, followed by 5-min wash at 80% B. Last, the column was equilibrated for 11 min at 2% B. At first, MS was operated in untargeted data-dependent acquisition mode. Full MS data were acquired in the Orbitrap from 150 to 1650 mass/charge ratio (*m*/*z*) with a 60,000 resolution at 200 *m*/*z* and 80-ms maximum injection time (IT). The auto gain control (AGC) target was set to a maximum of 3 × 10^6^ ions. An MS scan was followed by MS2 scans for the 10 most abundant precursor ions with 15,000 resolution at 200 *m*/*z* and 120-ms maximum IT. Each precursor was selected with an isolation window of 1.2 *m*/*z*, followed by fragmentation at a normalized collision energy (NCE) of 27%. The fragmented precursors were dynamically excluded for a duration of 20 s. In targeted mode, the MS data were acquired with 60,000 resolution at 200 *m*/*z* over the mass range from 150 to 1450 *m*/*z*, 3 × 10^6^ AGC target, and 25 ms maximum IT. In parallel reaction monitoring, MS2 data were acquired for the target peptides with parallel reaction monitoring scans using 60,000 resolution at 200 *m*/*z*. The target precursor list was provided with preselected charge states, the corresponding *m*/*z*, and collision energy values of 32% for the subdominant OVA epitope KVVRFDKL and 27% for all other target peptides. The normalized AGC target was set to 1000% (or 1 × 10^6^). The maximum IT mode was set to dynamic, allowing sampling of a minimum of five points across the chromatographic peak. The dynamic RT feature using the Pierce PRTC mixture was active. LC-MS data acquired in data-dependent acquisition mode were searched by using PEAKS Studio 10.6 software against UniProt mouse protein sequence data to which the sequence of chicken OVA (P01012) was added. Database search was performed using the following parameters: Parent and fragment mass error tolerance were set to 20 parts per million (ppm) and 0.05 Da, respectively; precursor mass search was set as monoisotopic; no enzyme was selected and the digestion mode was set to unspecific; 100 maximum missed cleavages were allowed; variable modifications were methionine oxidation and N-terminal acetylation; and a maximum of three posttranslational modifications were allowed. The targeted LC-MS data were analyzed with Skyline software (version 20.2) (*86*). Top six intensity product ions were extracted with 8-ppm mass tolerance. Detected peaks were manually curated, the MS2 data were first manually evaluated, and then the identification was further scored by comparison to high-quality fragmentation spectra (MS2) predicted for the corresponding peptide sequences by Prosit, a software based on deep learning (*87*). The evaluation of the similarity of the experimental and predicted MS2 spectra is based on the normalized spectral contrast angle (dotp) (*88*).

### Tumor mutanome analysis and T cell epitope prediction

Tumor exome and transcriptome sequencing was performed as described previously (*17*). For exome analysis and mutation calling of mouse samples, we used the Burrows-Wheeler aligner to map the Fastq files to the mouse reference genome (GRCm38). For read group addition and duplicate read marking, we used Picard (http://picard.sourceforge.net/). GATK was applied for subsequent steps of data preprocessing according to the Best Practices workflow. Last, somatic mutations were called using MuTect2 and annotated using ANNOVAR. Toward T cell epitope prediction, extraction of peptide sequences (21-mer) centered on the somatic single-nucleotide variant was performed by ANNOVAR. The peptide sequences were put into Fasta format and used as input for neoepitope prediction. The prediction was done using netMHCpan version 4.1b (https://services.healthtech.dtu.dk/service.php?NetMHCpan-4.1) for 8- to 10-mer binding to H-2K^b^ and H-2D^b^. The prediction results were manually curated for encompassing the mutated site and filtered for a predicted MHC affinity of <1 μM.

### Immunoprecipitation of MHC-bound peptides

Cells were lysed in 50 mM tris-Cl, pH 8.0, 150 mM NaCl, 5 mM EDTA, 0.5% Zwittergent 3-12 (*N*-dodecyl-*N*,*N*-dimethyl-3-ammonio-1-propanesulfonate), and protease inhibitor (Complete, Roche Applied Science) for 2 hours at 0°C (*89*). Lysates were successively centrifuged for 10 min at 2500*g* and for 45 min at 31,000*g* to remove nuclei and other insoluble materials, respectively. Next, lysates were passed through a CL-4B Sepharose column to preclear the lysate. The cleared lysate was passed through two columns in series containing anti–H-2K^b^ Ab (clone no. Y-3) coupled to protein A Sepharose and anti–H-2D^b^ Ab (clone no. 28-14-8) coupled to protein A Sepharose. The Ab columns were washed with lysis buffer, low-salt buffer (20 mM tris-Cl, pH 8.0, and 120 mM NaCl), high-salt buffer (20 mM tris-Cl, pH 8.0, and 1 M NaCl), and lastly, low-salt buffer. Peptides were eluted with 5 ml of 10% acetic acid and purified on a 10-kDa filter (Microcon, Millipore). The filtrate was diluted with 0.1% TFA and purified by solid-phase extraction (Oasis HLB, Waters) using 30% ACN in 0.1% TFA to elute the peptides.

### Immunopeptidome fractionation

Two-step fractionation was performed on an Easy-1000 LC system in combination with an Agilent 1200 series variable wavelength detector G1314B, set at 214 nm, in which a 50-nl capillary flow cell (LC-Packings) was installed, and controlled via the Agilent 1200 Infinity Series instant pilot G4208A. The fractionations were done with a 20 cm–by–200 μm inside diameter column, packed in-house with Reprosil Aqua C18, 3 μm, 120 Å (Dr. Maisch). The first fractionation was done with a gradient from 2% B to 50% B for 30 min (buffer A: 0.1% FA in water; buffer B: 20/80/0.1 water/ACN/FA) at a flow rate of 2 μl/min. Fractions were manually collected, directly from the exit capillary from the flow cell, into Eppendorf cups prefilled with 50 μl of water/ACN/0.1% FA as follows: initially, three 5-min fractions, followed by 30-s fractions until 33 min, followed by three 5-min fractions. The second fractionation was done in a similar fashion, but the column was run with a high-pH buffer system (buffer A: 10 mM ammonium bicarbonate in water, pH 8.4; buffer B: 20 mM ammonium bicarbonate in 80% ACN).

### MS of immunopeptidome

Samples were dissolved in water/FA (100/0.1, v/v) and analyzed by online C18 nano-HPLC MS/MS with a system consisting of an Ultimate3000nano gradient HPLC system (Thermo Fisher Scientific, Bremen, Germany) and an Exploris480 mass spectrometer (Thermo Fisher Scientific). Fractions were injected onto a cartridge precolumn (300 μm by 5 mm, C18 PepMap, 5 μm, 100 A) and eluted via a homemade analytical nano-HPLC column [50 cm by 75 μm; Reprosil-Pur C18-AQ 1.9 μm, 120 A (Dr. Maisch, Ammerbuch, Germany)]. The gradient was run from 2 to 36% solvent B (20/80/0.1 water/ACN/FA, v/v) for 120 min at 250 nl/min. The nano-HPLC column was drawn to a tip of around 10 μm and acted as the electrospray needle of the MS source. The mass spectrometer was operated in data-dependent top 20 MS/MS mode, with a higher-energy collisional dissociation collision energy at 30% and recording of the MS2 spectrum in the Orbitrap, with a quadrupole isolation width of 1.2 Da. In the master scan (MS1), the resolution was 60,000, and the scan range was 300 to 1500 at standard AGC target at maximum fill time of “standard.” A lock mass correction on the background ion *m*/*z* = 445.12003 was used. Precursors were dynamically excluded after *n* = 1 with an exclusion duration of 10 s and with a precursor range of 20 ppm. Charge states 1 (precursor selection range of 800 to 1500), 2 (precursor selection range of 400 to 800), and 3 (precursor selection range of 300 to 600) were included. For MS2, the first mass was set to 110 Da, and the MS2 scan resolution was 30,000 at an AGC target of ‘standard’@fill time of “auto”. In a postanalysis process, raw data were first converted to peak lists using Proteome Discoverer version 2.5 (Thermo Fisher Scientific) and then submitted to the UniProt mouse database (52,015 entries), supplemented with the custom proteome reference (data S9), using Mascot version 2.2.07 (www.matrixscience.com) for protein identification. Mascot searches were done with 10-ppm and 0.02-Da deviation for precursor and fragment mass, respectively, and no enzyme was specified. Methionine oxidation and cysteinylation of cysteine were set as variable modifications. The false discovery rate was set <1%, and in addition, peptides with mascot ion scores <35 were generally discarded. Candidate peptides specific, or clearly more abundant, in particular fractions and not the neighboring fractions were manually selected.

### Statistical analyses

Statistical tests are provided in the figure legends and in the corresponding Materials and Methods subsections for each analysis.
