# Supplementary material for: Dissection of the T cell infiltrate in mouse pancreatic tumors reveals an extensive and diverse tumor-reactive T cell repertoire
Source: Sci Adv. 2026 Apr 10;12(15):eadr6132. doi: 10.1126/sciadv.adr6132 (PMC13068058; doi:10.1126/sciadv.adr6132)
Supplement: Supplementary file 1 — Table S1 Figs. S1 to S12 Legends for data S1 to S16 [file sciadv.adr6132_sm.pdf]

Science Advances
AAAS

Supplementary Materials for

Dissection of the T cell infiltrate in mouse pancreatic tumors reveals an extensive and diverse tumor-reactive T cell repertoire

Hannes Kehm *et al.*

Corresponding author: Rienk Offringa, r.offringa@dkfz-heidelberg.de

Sci. Adv. **12**, eadr6132 (2026)
DOI: 10.1126/sciadv.adr6132

The PDF file includes:

Table S1
Figs. S1 to S12
Legends for data S1 to S16

Other Supplementary Material for this manuscript includes the following:

Data S1 to S16

DGE PDA TR-95	Rank	AUC	DGE ProjecTILs		DGE PDA NTR-57	Rank	AUC	DGE ProjecTILs
**Tnfrsf9**	1	0,8877	+		*Ly6a*	1	0,8136	-
**Il2rb**	2	0,8523	+		**Itgb7**	2	0,8235	+
**Lag3**	3	0,8224	+		**Gramd3**	3	0,7868	+
**Klrd1**	4	0,8116	+		**Gzmk**	4	0,7779	+
Tiprl	5	0,7899	-		**Cxcr3**	5	0,766	+
**Itgav**	6	0,785	+		**Ifi27l2a**	6	0,7549	+
**Hilpda**	7	0,7725	+		**Gpr18**	7	0,7434	+
**Fosl2**	8	0,7677	+		**Gzmb**	8	0,7316	+
**Klrc1**	9	0,764	+		**Cd7**	9	0,7316	+
**Stat3**	10	0,7532	+		*Gm26740*	10	0,7239	-
**Litaf**	11	0,7399	+		*Slfn2*	11	0,7195	-
**Rbpj**	12	0,7327	+		*Epsti1*	12	0,7102	-
Nap1l1	13	0,7314	-		**Emb**	13	0,7086	+
Ccrl2	14	0,7181	-		*Lef1*	14	0,7003	-
**Nr4a2**	15	0,7137	+					
Il21r	16	0,7096	-					
Tgfb1	17	0,7076	-					
Id2	18	0,7054	-					
Rac1	19	0,7048	-					
**Cst7**	20	0,7044	+					
Crem	21	0,7027	-					

Table S1. Discriminative power of individual genes comprised in TR95 and NTR57 gene signatures.

Evaluation of the power of the individual genes as included in the TR95 (left) and NTR57 (right) gene signatures with respect to discrimination between the tumor-reactive and bystander T cells in the scRNA-seq TIL data sets of 86 functionally tested CD8+ TCR clonotypes by means of ROC analysis. Genes with AUC > 0.7 are listed in order of power. See **Data S5** for AUC values of all genes. Genes that also segregate with, respectively, the TR or NTR state in the ProjecTILs scRNA-seq data set are highlighted (bold type). See **Data S6** for full analysis.

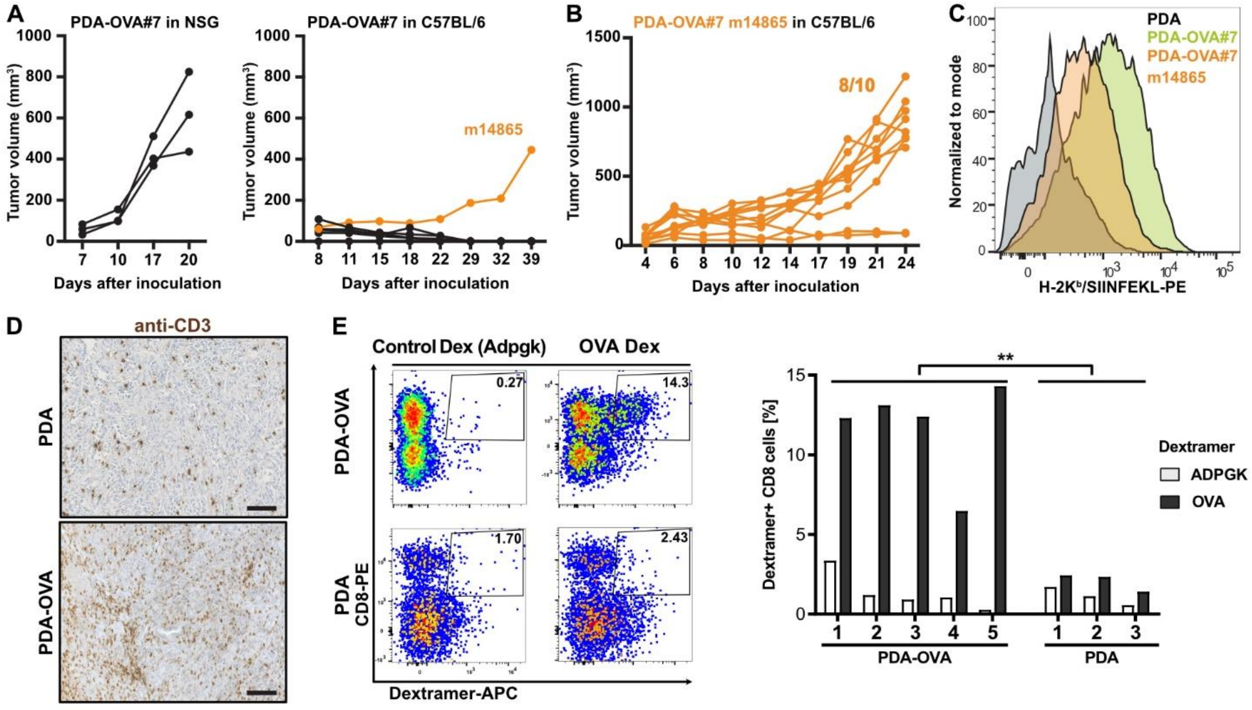

Fig. S1. Generation and characterization of PDA-OVA model.

(A) *In vivo* outgrowth of PDA_OVA#7 cells after s.c. injection into NSG (n=3) or C57BL/6 (n=5) mice (respectively 2x and 5x 10E6 cells/mouse). While all NSG mice developed tumors (left), this occurred in only one of the C57BL/6 mice (m14865) (right). **(B)** From this tumor, a new cell line was established (PDA-OVA#7_m14865), capable of efficient *in vivo* outgrowth upon s.c. injection (2x 10E6 cells/mouse) into immunocompetent C57BL/6 mice (n = 10). **(C)** H-2K^b/SIINFEKL expression at the surface of tumor cell line PDA-OVA#7_m14865 (hereafter referred to as PDA-OVA), as compared to the PDA-OVA#7 and parental PDA cell lines. **(D)** Increased CD3+ T-cell infiltration in PDA-OVA tumors as compared to parental PDA tumors, as detected by immunohistochemistry. Scale bar = 250 µm. **(E)** Flow cytometric detection of OVA-SIINFEKL-specific CD8+ T-cells in enzymatically dissociated PDA-OVA tumors by means of H-2K^b/SIINFEKL-specific dextramers. Dissociated PDA tumors and H-2K^b/Adpgk dextramers serve as controls. Representative flow cytometry plots (left) and data from multiple tumors (right) are shown. Two-sided Student's t-test. p-value ** < 0.01.

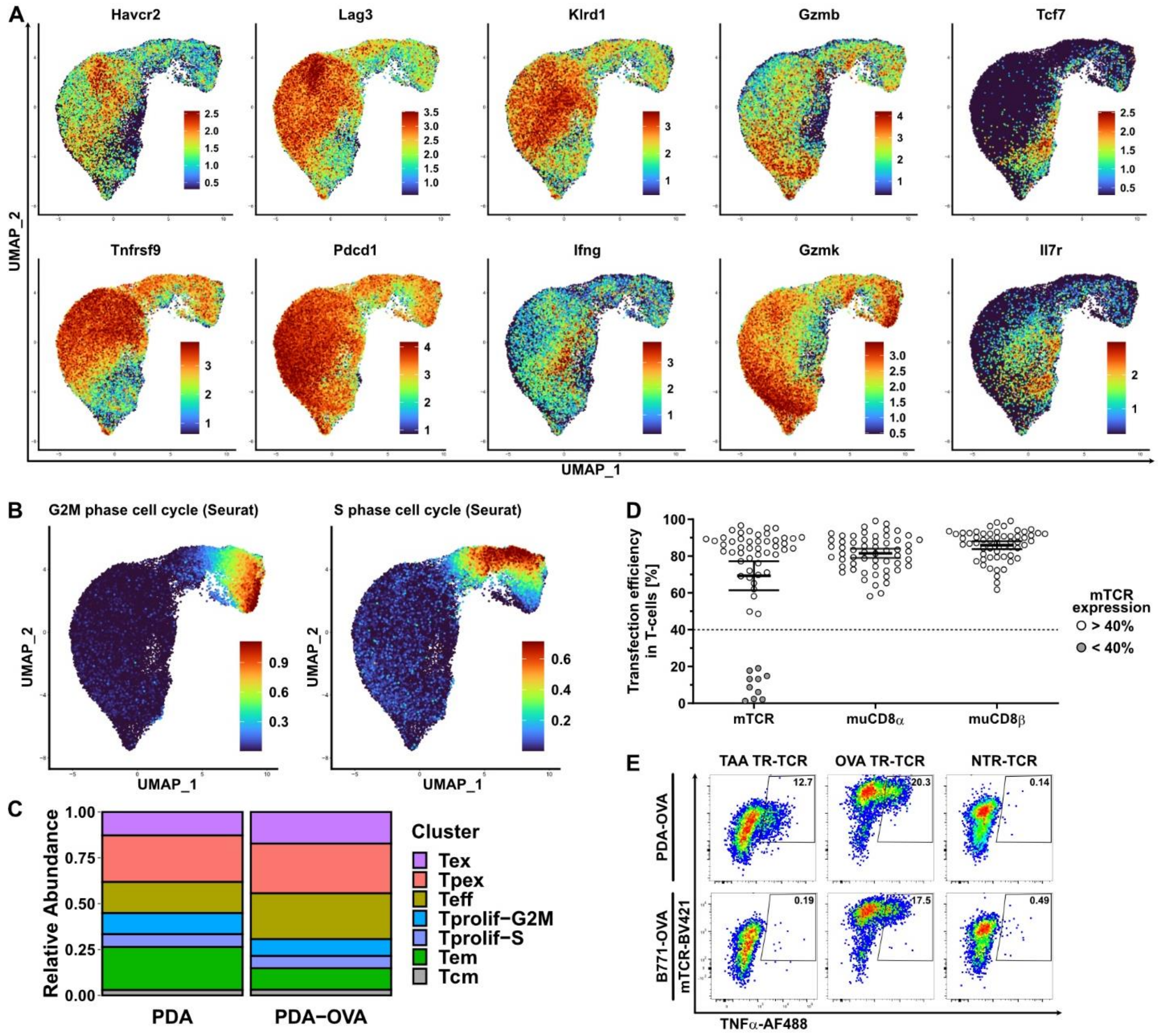

Fig. S2. Identification of CD8+ T-cell subsets based on expression of differentially expressed genes

(A) UMAPs of combined PDA and PDA-OVA CD8+ T-cell data sets illustrating the differences in expression of commonly described CD8+ T-cell exhaustion, effector, and stem-cell markers between T-cells residing in the clusters as defined in Fig. 2c. **(B)** Identification of proliferating cells in G2M-phase (left) or S-phase (right) using *CellCycleScoring* function in *Seurat*. **(C)** Comparison of the relative distribution of the T-cells over the different transcriptional states between PDA and PDA-OVA tumors. **(D)** Expression efficiency of murine T-cell receptors, as well as murine

CD8α and CD8β co-receptors upon co-transfection into human T-cells. A 40% transfection rate was set as a threshold for conclusive results in functional analyses. For ten TCRs, the expression efficiency was consistently lower, leading to their exclusion from further analyses. Mean ± 95% confidence interval displayed. **(E)** FACS data showing examples of the two main classes of TR-TCRs as isolated from PDA-OVA tumors. OVA-reactive TCRs (e.g. CT10.4) mediate T-cell reactivity against PDA-OVA as well as B771-OVA cells, whereas TR-TCRs (e.g. CT9.2) targeting non-OVA TAAs only react against PDA-OVA. NTR-TCRs (e.g. CT14.6) are negative controls that do not detect either cell line.

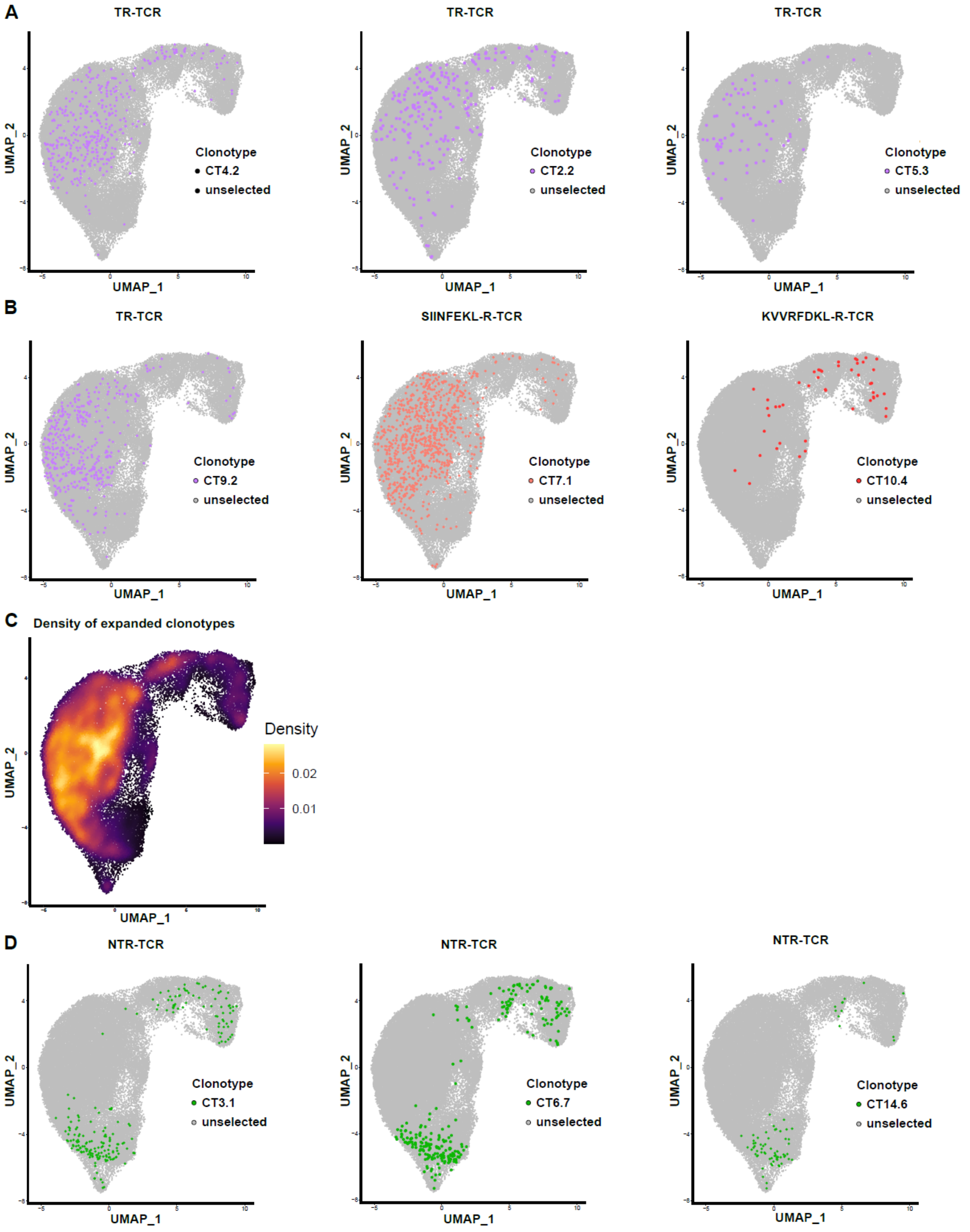

Fig. S3. Visualization of transcriptional states of T-cells comprised within single TCR-clonotypes

(A) UMAP projection of single TCR clonotypes showing representative examples of TAA-reactive clonotypes isolated from PDA tumors. **(B)** Same analysis as in panel A for TCR clonotypes isolated from PDA-OVA tumors detecting a TAA (left), the OVA/SIINFEKL immune-dominant epitope (center), or the subdominant OVA/KVVRFDKL epitope. **(C)** UMAP density plot showing the distribution of T-cells belonging to highly expanded CD8+ TCR clonotypes. **(D)** Same analysis as in panel A for T-cells belonging to three representative NTR-TCR clonotypes.

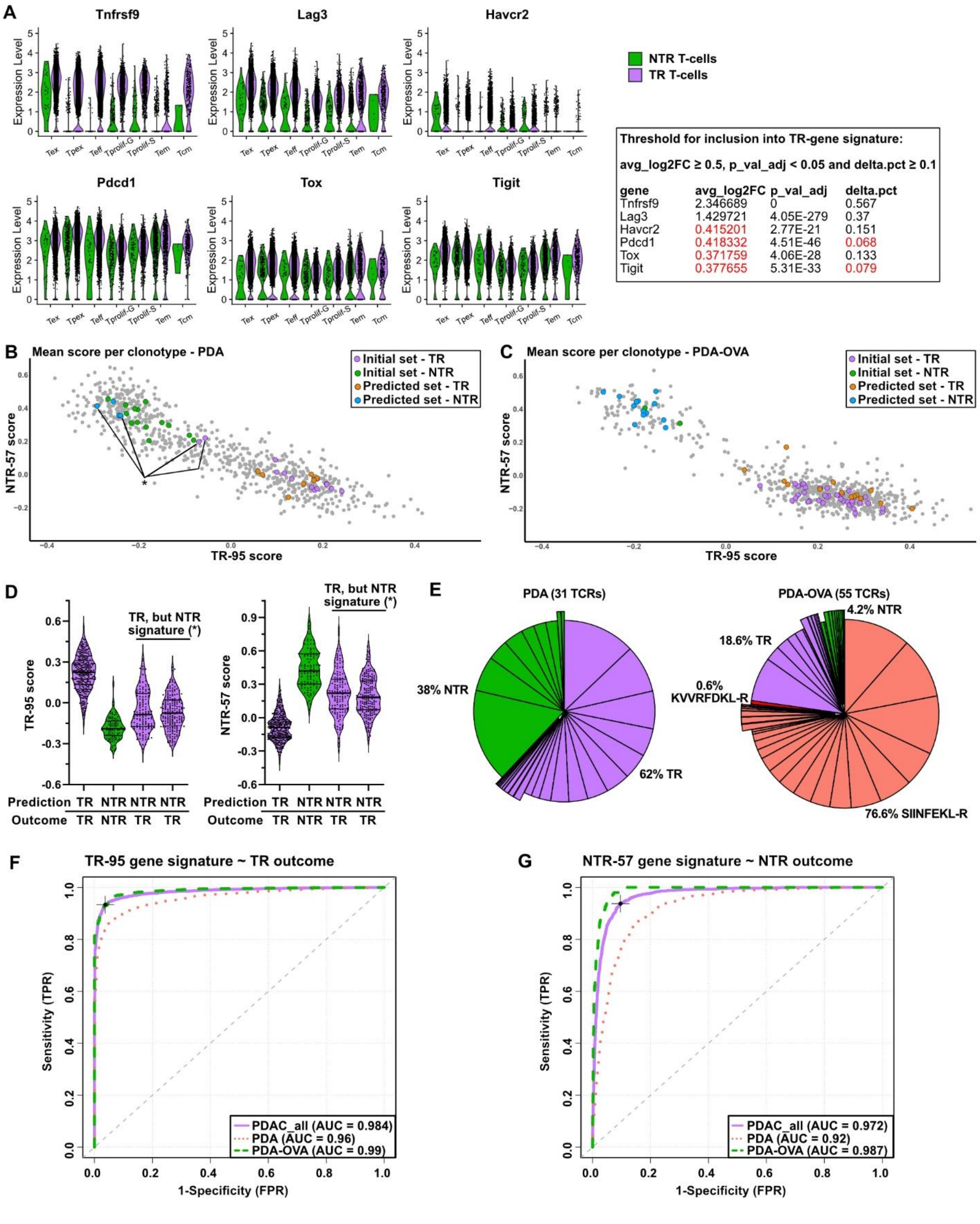

Fig. S4. Definition and validation of the TR-95 and NTR-57 gene signatures

(A) Violin plots comparing the expression of commonly described T-cell exhaustion genes within the PDA/PDA-OVA data set for all T-cells comprised within the functionally validated TR- versus NTR-TCR clonotypes. The box indicates thresholds for inclusion into the TR-gene signature as differential gene expression between cells of TR/NTR specificity exceeding avglog2FC ≥ 0.5, p_val_adj < 0.05 and delta.pct ≥ 0.1 (for details see **Data S3**). **(B)** 2D-matrix depicting the mean TR-95 and NTR-57 scores of each of the CD8+ TCR clonotypes comprised in the PDA data set. Clonotypes with at least 3 cells are shown. Highlighted in different colors are the 19 predominant clonotypes that were included in the first functional screen (magenta for TR, green for NTR clonotypes), as well as the 12 further clonotypes included in the second screen that were predicted by means of the TR-95/NTR-57 gene signatures as TR (orange) or NTR (blue). The * marking indicates clonotypes that, based on gene signature, were predicted to be NTR, but revealed anti-tumor activity in functional testing *in vitro*. **(C)** Same analysis as in panel B for the clonotypes comprised in the PDA-OVA data set. Highlighted in different colors are the 28 predominant clonotypes that were included in the first screen (magenta for TR, green for NTR clonotypes), as well as the 27 further clonotypes that were predicted by means of the TR-95/NTR-57 gene signatures as TR (orange) or NTR (blue). **(D)** Violin plots showing the TR-95 (left) and NTR-57 (right) signature scores for individual T-cells belonging to a typical TR clonotype (CT5.3, magenta), a typical NTR clonotype (CT1.4, green), and two unusual clonotypes (CT1.1, CT2.5, magenta) the TCR of which mediated anti-tumor reactivity *in vitro* in spite of displaying a NTR signature (magenta dots marked with asterisks in panel B). Mean ± 95% confidence interval displayed. **(E)** Overview of the functionally characterized outcome of the combined 86 TCRs from PDA (left) and PDA-OVA (right), grouped by outcome. Exploding parts of the pie chart represent TCRs included in the second functional screen that were predicted using the TR-95/NTR-57 gene signatures. **(F)** Receiver operating characteristic (ROC) analysis predicts the accuracy of the TR-95 gene signature for describing the tested tumor-reactive outcome of T-cells in the complete PDA/PDA-OVA data set (PDAC_all), or on PDA and PDA-OVA subsets separately. **(G)** Same analysis as in panel F but for the NTR-57 gene signature.

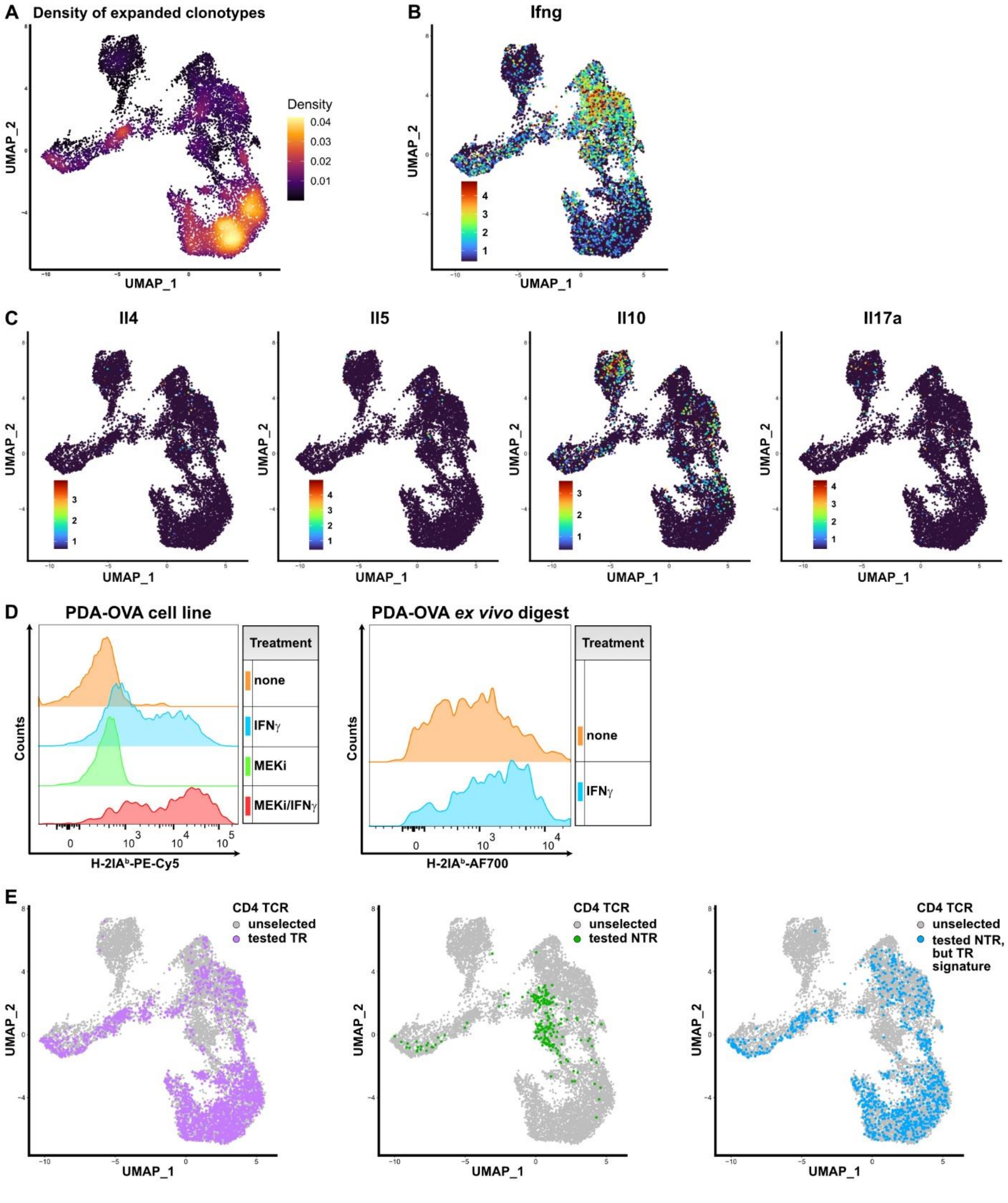

Fig. S5. Dissection of the MHC class II-restricted CD4+ T-cell immunity in murine PDA tumors (Part 1)

(A) Density plot highlighting the enrichment of expanded CD4+ clonotypes on the UMAP based on clonal expansion. **(B, C)** Density plots highlighting the expression of the indicated cytokines. **(D)** Left panel: cell surface expression of MHC class II I-A^b on PDA-OVA cells cultured under routine conditions or after pre-treatment with IFNγ/MEK inhibitor GDC-0623 alone, or when combined. Right panel: cell surface expression of MHC class II I-A^b on PDA-OVA tumor cells in freshly dissociated tumor tissue, analyzed without and with IFNγ pre-treatment. The co-expression of OVA and GFP in PDA-OVA cells (see Methods) was used to set an unambiguous gate on the $CD45^-$ GFP^+ tumor cells. **(E)** UMAP projection of the individual T-cells comprised by the 17 CD4+ clonotypes with a TR signature expressing TCRs that were confirmed TR in the *in vitro* assays (left), comprised by the 8 CD4+ clonotypes expressing an NTR signature that were confirmed to be NTR (middle), or comprised by the 7 clonotypes expressing a TR signature, but found to be NTR upon testing *in vitro* (right).

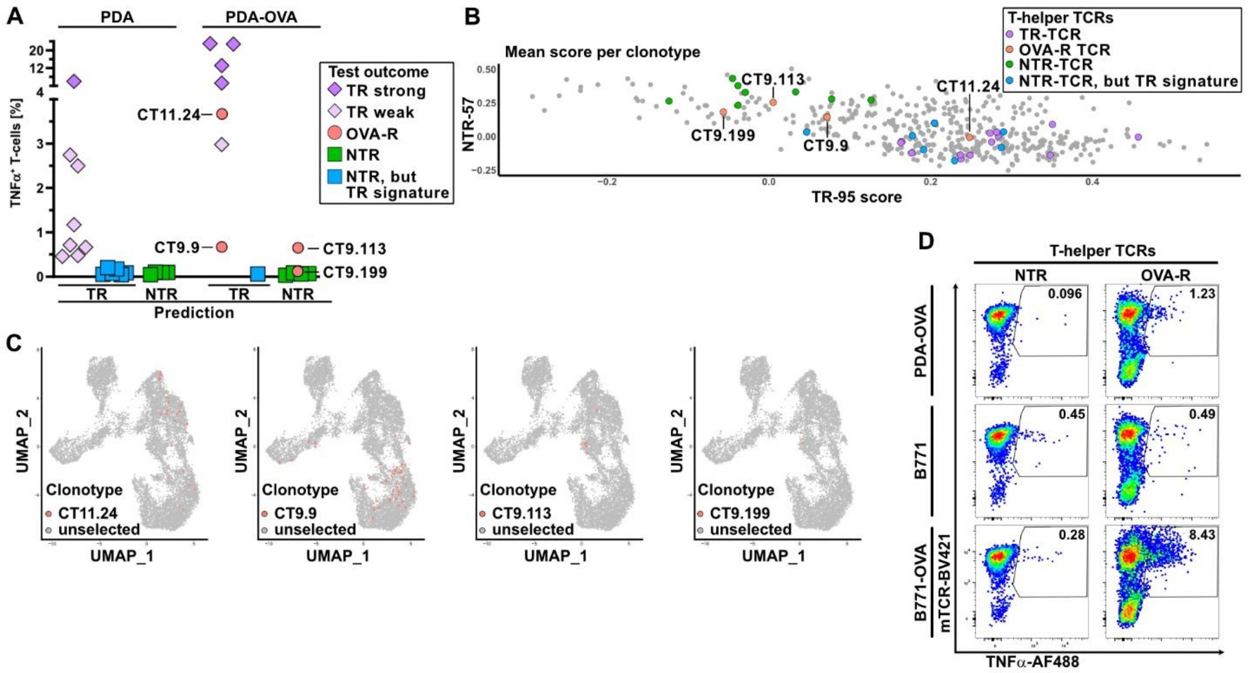

Fig. S6. Dissection of the MHC class II-restricted CD4+ T-cell immunity in murine PDA tumors (Part 2)

(A) Version of Fig. 5E focusing on the 4 OVA-reactive clonotypes isolated from PDA-OVA tumors, showing the relationship between gene signature and *in vitro* recognition of PDA-OVA cells. **(B)** Version of Fig. 5G focusing on the aforementioned OVA-reactive clonotypes showing the TR and NTR scores resulting in a TR prediction for clonotypes CT11.24 and CT9.9, and a NTR prediction for clonotypes CT9.113 and CT9.199. **(C)** UMAP projection of each of these clonotypes, confirming that the TR-prediction of CT11.24 and CT9.9, and the NTR-prediction of CT9.113 and CT9.199 properly reflect the distribution of the T-cells in, respectively, the Tex/Tpex and Tem clusters. **(D)** Representative data of functional TCR screening experiments showing the TNFα production of T-cells expressing an NTR (CT12.89) or an OVA-specific CD4+ TCR (CT9.113). T-cell reactivity is measured against PDA-OVA cells, B771 B-lymphoma cells or such cells expressing full-length OVA antigen (B771-OVA).

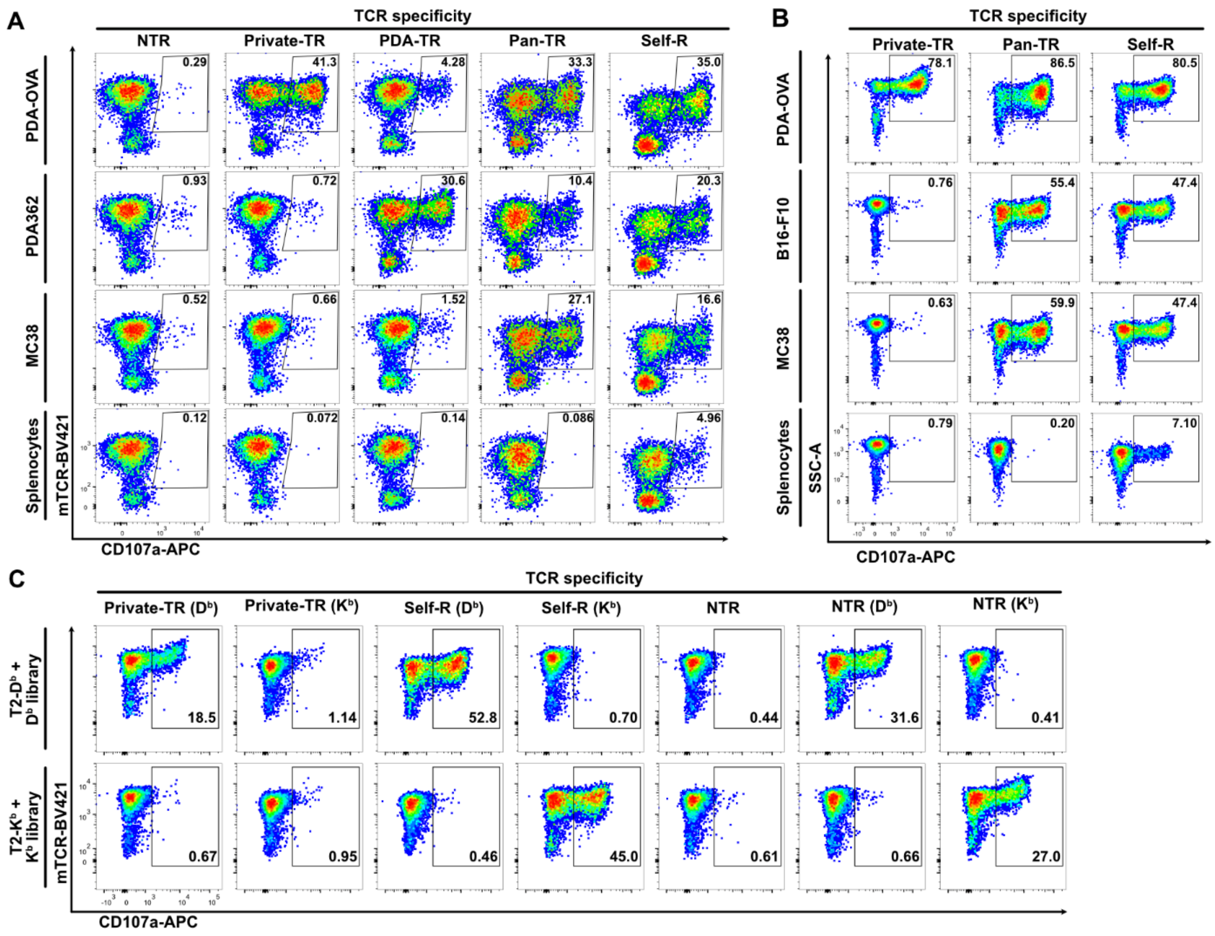

Fig. S7. Differentiation between CD8+ TR-TCRs based on tumor-reactivity and MHC-restriction.

(A) Representative data of functional TCR screening experiments showing the CD107a surface expression of T-cells expressing different classes of TR-TCRs isolated from CD8+ clonotypes. From left to right: an NTR-TCR (CT12.37); a private TR-TCR (CT11.2) reactive to PDA-OVA only; a PDA-reactive TCR (CT2.5) reactive against PDA, PDA-OVA and PDA30362; a pan TR-TCR (CT14.2) also responding to MC38 cells (as well as against other tested C57BL/6-derived tumor cell lines of H-2^b origin B16-F10 and B771); and a self-R TCR (CT11.10) that in addition detects a self-antigen expressed by cultured C57BL/6 mouse splenocytes. See Fig. 6A for measurement of T-cell reactivity in the same experiment on the basis of TNFα production. **(B)** Additional representative data of functional TCR screening experiments showing the reactivity of T-cells expressing

different CD8+ TR-TCRs (from left-to-right CT14.7, CT14.2, CT11.10) in co-culture against different C57BL/6 tumor cell lines, in particular PDA-OVA, B16-F10 and MC38, as well as normal C57BL/6 splenocytes. **(C)** Reactivity of T-cells expressing TCRs with the indicated antigen/tumor specificity (from left-to-right CT14.7, CT11.36, CT11.10, CT2.6, CT12.17, CT11.39, CT7.6) upon incubation with T2-K^b or T2-D^b cells that were pulsed with synthetic H-2K^b-motif and H-2D^b-motif peptide libraries, respectively.

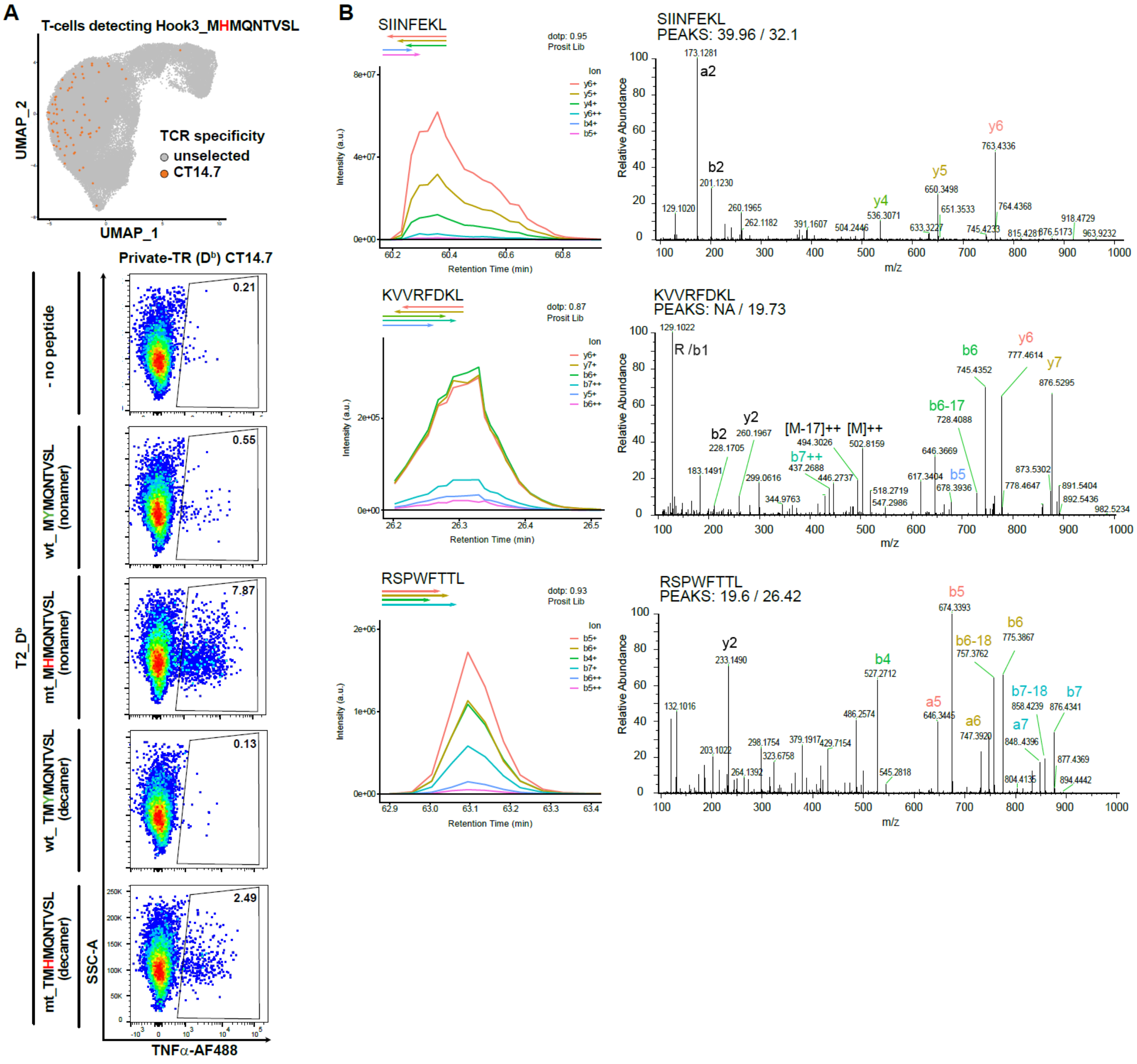

Fig. S8. Differentiation between CD8+ TR-TCRs based antigen specificity.

(A) (Top) UMAP projection of T-cells encompassed by tumor-reactive clonotype CT14.7 expressing the H-2D^{b}-restricted TCR targeting the mutated Hook3 neo-epitope. (Bottom) Flow cytometry data shows specific detection of mutated nona-/decamers (red H amino acid), but not wild-type variants (green Y amino acid, bottom) of the Hook3 epitope as pulsed onto T2-D^{b} cells. **(B)** Fragment-ion chromatograms are shown for the indicated peptides detected by targeted mass spectrometry in MAE immunopeptidome isolates at high resolution and high mass accuracy. Different colors indicate the top 6 extracted peptide fragments (ions). The nature of the fragment-ions is indicated by the arrows under the peptide sequences (left-to-right and its

opposite indicate b- and y-ions from the N- and C-termini, respectively). The dotp is indicated for each peptide. (right-hand panels) High-resolution fragmentation spectra are shown for the indicated peptides. Different fragment-ions are shown in different colors. Additional fragment-ions that are not monitored are indicated in black. For each spectrum, the peptide sequence is shown with the score underneath for its identification by PEAKS database search. The scores separated by a slash indicate 2 independent detections. The peptide with the lowest abundance was only detected in the targeted LC-MS analysis.

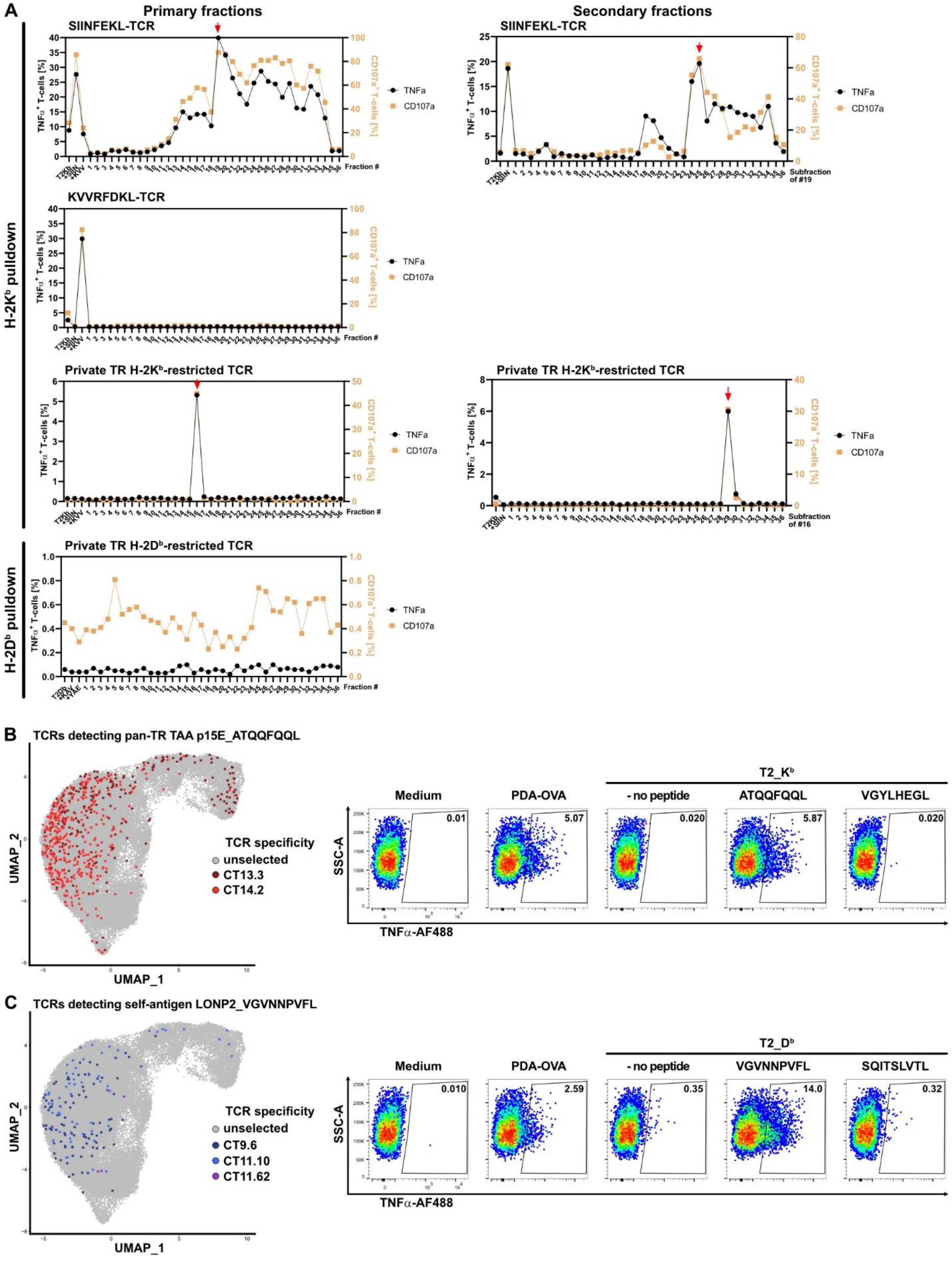
A
Primary fractions
Secondary fractions
H-2K^b pulldown
H-2D^b pulldown
SIINFEKL-TCR
KVVRFDKL-TCR
Private TR H-2K^b-restricted TCR
Private TR H-2D^b-restricted TCR
TNFα⁺ T-cells [%]
CD107a⁺ T-cells [%]
TNFa
CD107a
Fraction #
Subfraction of #19
Subfraction of #16
B
TCRs detecting pan-TR TAA p15E_ATQQFQQL
TCR specificity
unselected
CT13.3
CT14.2
UMAP_1
UMAP_2
T2_K^b
Medium
PDA-OVA
- no peptide
ATQQFQQL
VGYLHEGL
0.01
5.07
0.020
5.87
0.020
SSC-A
TNFα-AF488
C
TCRs detecting self-antigen LONP2_VGVNNPVFL
TCR specificity
unselected
CT9.6
CT11.10
CT11.62
UMAP_1
UMAP_2
T2_D^b
Medium
PDA-OVA
- no peptide
VGVNNPVFL
SQITSLVTL
0.010
2.59
0.35
14.0
0.32
SSC-A
TNFα-AF488

Fig. S9. Immunopeptidomics-based identification of a pan-TR and a self-antigen.

(A) Representative examples of further screening outcome of primary (left) and secondary (right) HPLC-fractions with T-cells expressing tumor-reactive TCRs, in particular (top to bottom): OVA/SIINFEKL-specific H-2K^b-restricted TCR CT7.1, OVA/KVVRFDKL-specific H-2K^b-restricted TCR CT10.4, private TR H-2K^b-restricted TCR CT11.40 and private TR H-2D^b-restricted TCR CT11.2. Whereas the immunodominant OVA/SIINFEKL peptide was readily detected by LC-MS-MS analysis in secondary fraction 25, the subdominant OVA/KVVRFDKL was not detectable even after the primary fractionation (only synthetic peptide renders a positive signal). For the private TR H-2K^b-restricted TCR we did obtain a clearly positive secondary fraction, but the epitope could not be resolved by LC-MS-MS. For the private TR H-2D^b-restricted TCR we obtained a weak signal in the primary fraction screening, but this was insufficiently selective towards proceeding with secondary fractionation. Peptide fractions were loaded onto T2 cells expressing either H-2K^b or H-2D^b, matching the pre-defined MHC restriction of the TCR concerned. **(B)** (Left) UMAP projection of T-cells encompassed by tumor-reactive clonotypes CT13.3 and CT14.2 expressing H-2K^b-restricted TCRs targeting the p15E-derived epitope. (Right) Flow cytometry data showing specific detection of synthetic peptide ATQQFQQL, but not of a control peptide as identified in secondary-fraction H-2K^b#12-19, as pulsed onto T2-K^b cells. **(C)** (Left) UMAP projection of T-cells encompassed by tumor-reactive clonotypes CT9.6, CT11.10 and CT11.62 expressing H-2D^b-restricted TCRs targeting the LONP2-derived epitope. (Right) Flow cytometry data showing specific detection of synthetic peptide VGVNNPVFL, but not of a control peptide as identified in secondary fraction H-2D^b#19-27, as pulsed onto T2-D^b cells. Control peptides are, respectively, VGYLHEGL, a H-2K^b-binding peptide derived from U5 small nuclear ribonucleoprotein 200 kDa helicase, a spliceosome subunit, and SQITSLVTL, a H-2D^b-binding peptide derived from origin recognition complex subunit 5, a protein involved in DNA replication.

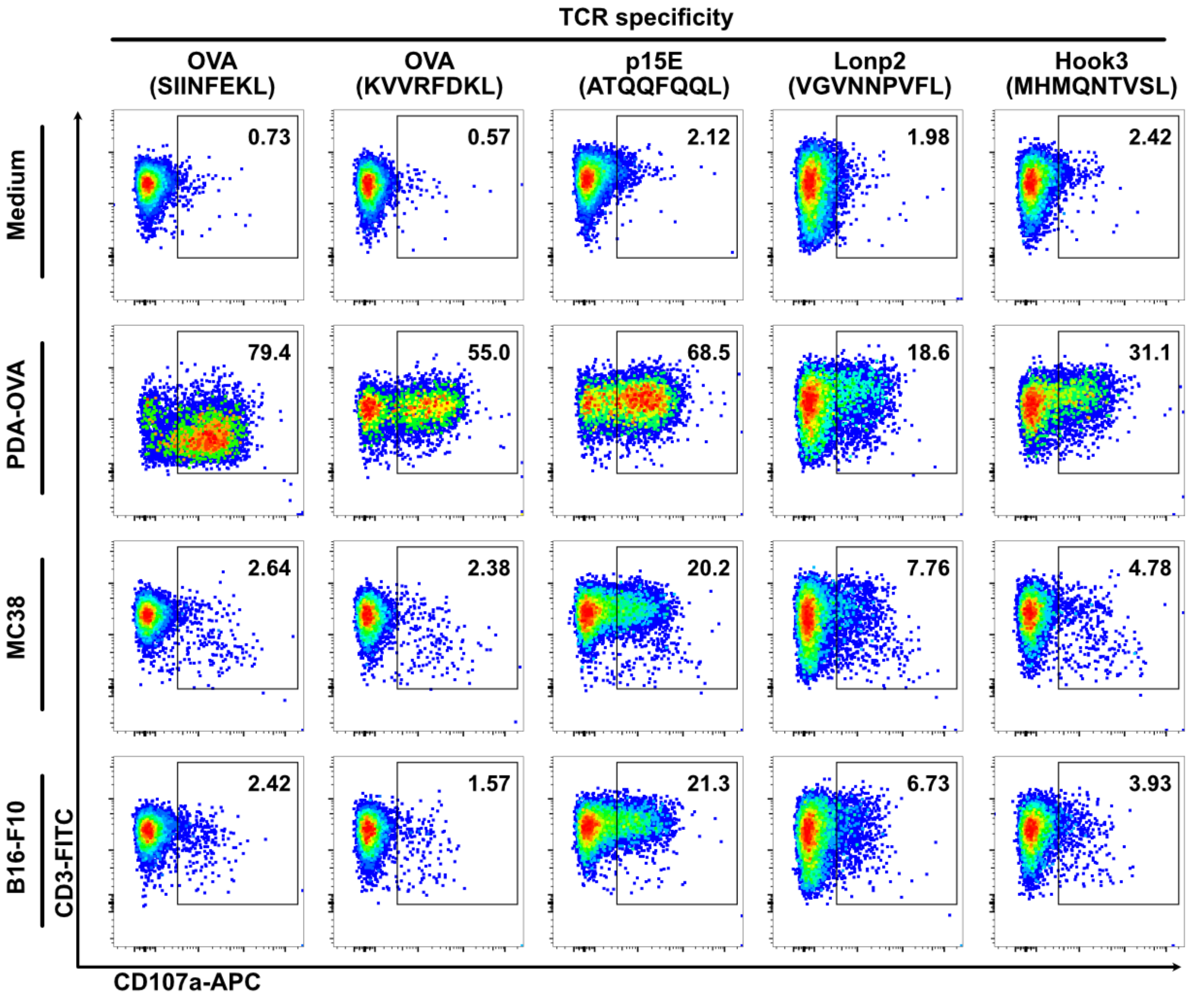

Fig. S10. Reactivity of tumor-reactive TCRs in retrovirally-transduced primary mouse T-cells.

(A) Representative flow cytometry data showing in vitro reactivity of TCR-transduced T-cells expressing tumor-reactive (TR) or non-tumor-reactive (NTR) TCRs in the presence of the indicated tumor cells. Shown are data for the TCRs derived from the following clonotypes, as listed in **Data S10**: CT7.1, CT10.4, CT14.2, CT9.6, CT14.7

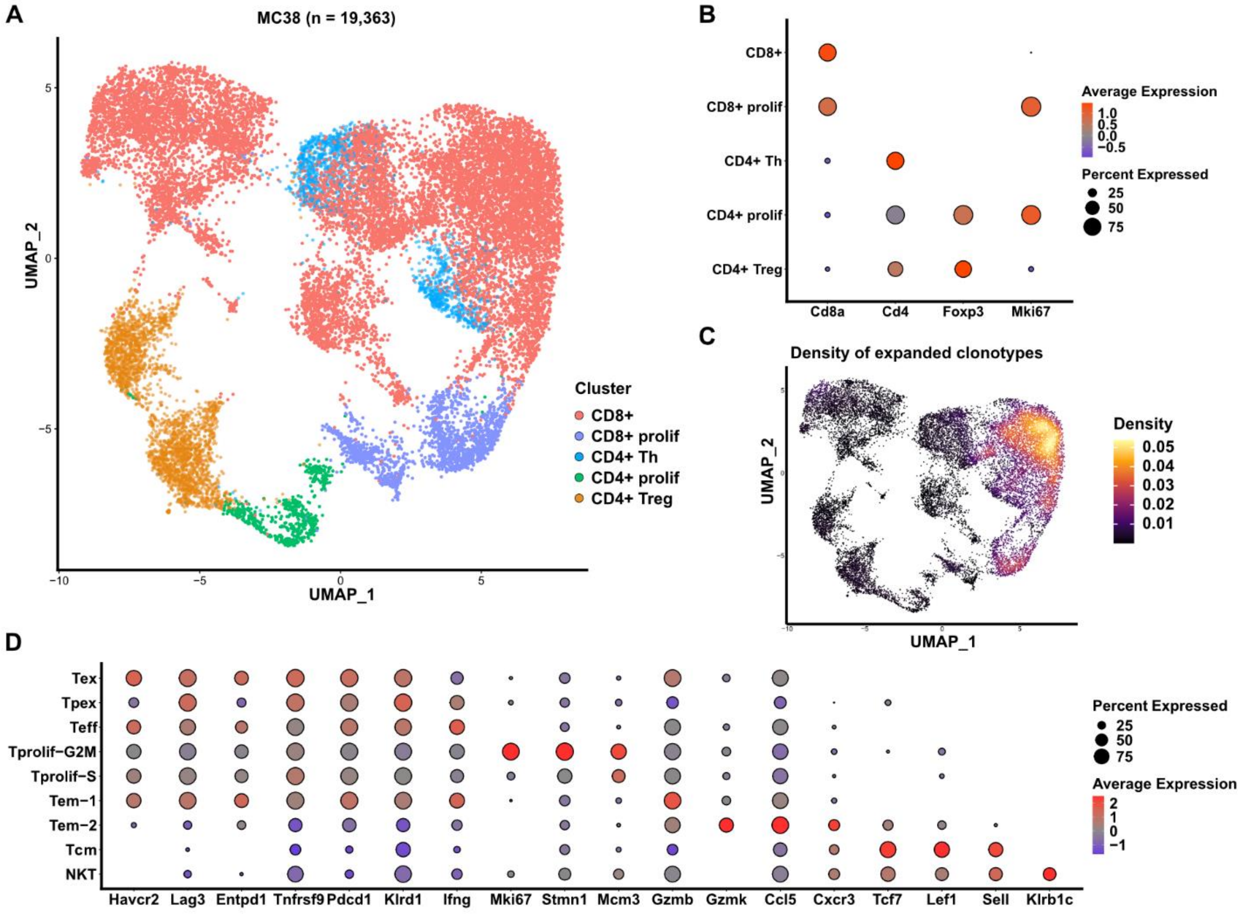

Fig. S11. Charting of the T-cell response in the MC38 tumor model.

(A) UMAP of 19,363 T-cells isolated from 3 MC38 tumors, clustered into major T-cell subtypes. **(B)** Expression of T-cell lineage markers displayed in dot plot (see **Data S13** for complete gene lists). **(C)** UMAP density plot showing the distribution of T-cells belonging to highly expanded CD8+ TCR clonotypes. **(D)** Differentially expressed genes reflecting the 9 main transcriptional states as identified in the CD8+ T-cell subset (see **Data S14** for complete gene lists).

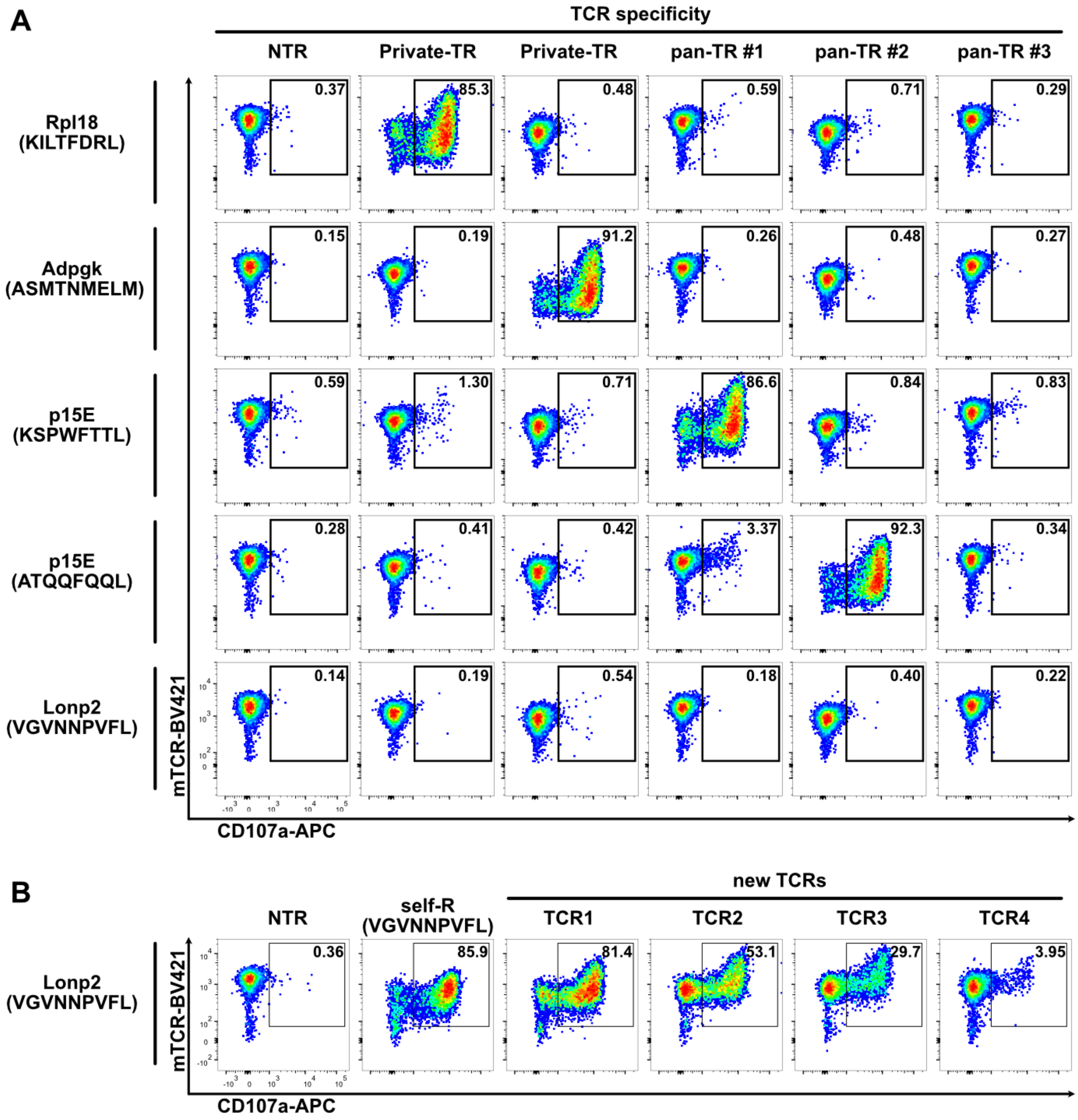

Fig. S12. Differentiation of MC38 and PDA tumor-derived CD8+ TR-TCRs based on tumor-reactivity and antigen specificity.

(A) Example of representative flow cytometry data showing in vitro reactivity of TCR-transduced T-cells expressing MC38-derived tumor-reactive (TR) or non-tumor-reactive (NTR) TCRs in the presence of the indicated synthetic peptide epitopes. Shown are data for the TCRs derived from the following clonotypes, as listed in **Data S15**: CT17.26, CT17.1, CT15.33, CT15.17, CT15.8, CT15.36 **(B)** Flow cytometry data of TCRs selected based on sequence homology against the LONP2/VGVNNPVFL epitope. Shown are data for

the TCRs derived from the following clonotypes, as listed in **Data S10/S16**: CT12.17, CT9.6, CT7.52, CT13.32, CT10.22, CT13.14

Data S1. Differentially expressed genes between clusters in T-cell subsets derived from PDA and PDA-OVA tumors

Data S2. Differentially expressed genes between clusters in CD8 T-cell subsets derived from PDA and PDA-OVA tumors

Data S3. Mutanome analysis using MuTect2 in PDA tumor model

Data S4. Differentially expressed genes between TR and NTR T-cells for the 48 TCRs initially tested based on size from PDA and PDA-OVA tumors, including genes selected for TR_95 and NTR_57 gene sets

Data S5. TR_95 and NTR_57 score averaged for all cells per clonotype in the CD8 data set

Data S6. Investigation of single-gene impact with outcome of all cells of total dataset of 86 TCRs using receiver operating characteristics analysis; evaluation of overlap between TR-95 and NTR-57 gene sets with gene signatures reflecting T-cell states as defined in ProjecTILs, as well as TR/NTR gene sets as defined in our prior study in human PDAC.

Data S7. Differentially expressed genes between Tex/Tpex/Teff versus Tem clusters in mouse ProjecTILs tumor data set

Data S8. Differentially expressed genes between clusters in CD4 T-cell subsets derived from PDA and PDA-OVA tumors

Data S9. TR_95 and NTR_57 score averaged for all cells per clonotype in the CD4 data set

Data S10. Overview of tested TCR clonotypes in PDA model

Data S11. Mutanome-based identification of private H-2Db-restricted PDA-OVA Hook3 epitope

Data S12. Custom proteome reference used for spectra matching of tandem mass spectrometry

Data S13. Differentially expressed genes between clusters in T-cell subsets derived from MC38 tumors

Data S14. Differentially expressed genes between clusters in CD8 T-cell subsets derived from MC38 tumors

Data S15. Overview of tested TCR clonotypes in MC38 model

Data S16. Overview of TCR clonotypes tested for Lonp2 reactivity
